# Accurately Tunable AuNC‐ZIF Content Architecture Based on Coordination‐Dissociation Mechanism Enables Highly Brightness Dual‐Site Fluorescent Biosensor

**DOI:** 10.1002/advs.202408400

**Published:** 2024-12-04

**Authors:** Junyang Chen, Yuqian Wang, Runpu Shen, Wei Li, Sainan Gao, Zhikang Xiao, Qiyan Lv, Xiaojie Song, Jianzhong Xu, Gaoxiang Xu, Huifang Cui, Zhaohui Li

**Affiliations:** ^1^ School of Life Sciences Zhengzhou University Zhengzhou Henan 450001 China; ^2^ School of Chemistry and Chemical Engineering Shaoxing University Shaoxing Zhejiang 312000 China; ^3^ School of Basic Medical Sciences Zhengzhou University Zhengzhou Henan 450001 China; ^4^ Department of Orthopedics The First Affiliated Hospital of Zhengzhou University Zhengzhou Henan 450052 China; ^5^ College of Chemistry, Institute of Analytical Chemistry for Life Science Zhengzhou University Zhengzhou Henan 450001 China

**Keywords:** biosensors, carbon dots (CDs), dual‐site fluorescence, gold nanoclusters, zeolitic imidazolate frameworks (ZIFs)

## Abstract

The quantum yield and fluorescence intensity of gold nanocluster (AuNC) nanocarriers are critical parameters for developing ultrasensitive biosensors. In this study, AuNCs‐zeolitic‐imidazolate‐framework (Au‐ZIF) nanocomposites are systematically constructed by impregnating AuNCs onto the ZIF‐8 surface through a coordination‐dissociation mechanism, resulting in a dual‐site fluorescence‐loaded structure. In this configuration, AuNCs are anchored to the external surface while the integrity of the inner cavity remains intact. The surface of ZIF‐8 induces a confinement effect on the configuration and electrons of AuNCs, significantly enhancing luminescence (18‐fold increase). The quantum yield of AuNCs exhibits an increase of more than 13‐fold, from 2.80% to 38.1%. This approach demonstrates broad applicability and maintains strong fluorescence across different ZIFs. Additionally, a novel nanocomposite, Au‐ZIF@carbon‐dots (CDs), is synthesized by encapsulating CDs into the inner cavity of Au‐ZIF. A ratiometric fluorescence detection platform is subsequently developed and incorporated into hydrogels for the quantitative detection of the pesticide triazophos. By employing an image‐processing algorithm, quantitative detection is achieved with a detection limit of 0.07 ng mL⁻^1^. The findings provide crucial insights into the relationship between the assembly and performance of AuNCs and ZIFs, offering guidance for designing ultrasensitive multifunctional biosensors applicable in the field of biosensing.

## Graphical abstract

The precise construction of gold nanoclusters (AuNCs) and zeolite imidazolate framework‐8 (ZIF‐8) is achieved through the coordination‐dissociation mechanism, which enables the external loading of fluorescent substances while preserving the internal loading function, resulting in the construction of a high‐brightness dual‐site fluorescent probe that can be generalized to other materials. The probe successfully enables the highly sensitive detection of pesticides.

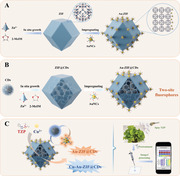

## 1 Introduction

As awareness of the importance of safety and health continues to grow, the necessity for immediate, direct, and precise monitoring of food, the environment, and bodily conditions also increases. To address this need, biosensors have been developed to significantly enhance the convenience, efficiency, and cost‐effectiveness of analytical detection across various applications and environments.^[^ ^1^ ^]^ Fluorescent metal nanoclusters, known for their unique physicochemical properties and notable optical characteristics, are recognized as a promising class of fluorescent nanomaterials for biosensing applications, attracting substantial interest.^[^ ^2^ ^]^ Gold nanoclusters (AuNCs), in particular, have gained ongoing attention due to their low toxicity, simple synthesis process, and favorable biocompatibility.^[^ ^3^, ^4^, ^5^, ^6^, ^7^ ^]^ Nevertheless, AuNCs generally exhibit lower luminescence efficiency compared to other fluorophores, such as carbon dots (CDs) and quantum dots, which has significantly restricted their practical applications.^[^ ^8^ ^]^ However, the inherent properties of AuNCs offer the potential forfluorescence efficiency enhancement through the restriction of intermolecular or intramolecular movement, providing innovative strategies for the rational design of advanced fluorescent composites.^[^ ^9^, ^10^ ^]^ Two principal approaches are generally employed to enhance the luminescence efficiency of nanomaterials based on AuNCs. The first approach involves restraining intramolecular movement using metal cations, organic solvents, and nanogels, which facilitate the activation of AIE properties in AuNCs.^[^ ^11^, ^12^, ^13^ ^]^ However, the potential biotoxicity of these chemicals and the unpredictable variation in luminescence intensity with changes in pH considerably limit their biological applications. To address this limitation, a second approach that uses host nanomaterials capable of loading AuNCs is considered a more practical alternative.^[^ ^14^, ^15^ ^]^ Through the selection of specific nanomaterial types as carriers, AuNCs can be rationally assembled to achieve greater luminescence efficiency through significant aggregation and restricted intramolecular movement, leading to enhanced fluorescence intensity. Furthermore, the chosen host nanomaterials can impart additional properties that extend beyond the characteristics of the individual components.

**Figure 1:**
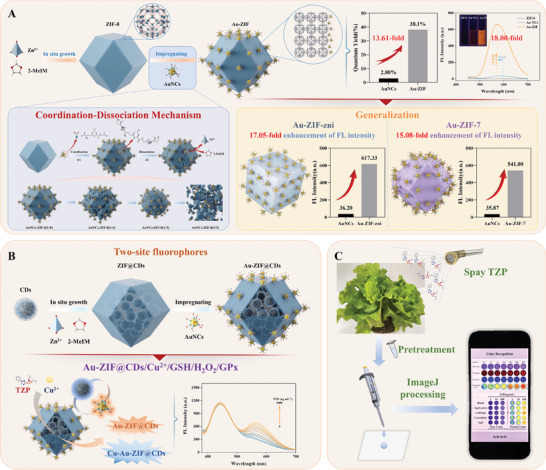
Schematic illustration of the precise and tunable synthesis, broad generalization potential, dual‐site fluorescent construction, and sensing application of the Au@ZIF Component. A) The accurate synthesis of AuNCs@ZIF Content architecture was achieved by impregnating AuNCs onto the ZIF‐8 surface via a coordination‐dissociation mechanism, significantly enhancing the quantum yield and fluorescence intensity of AuNCs. This strategy is also easily adaptable to other ZIFs. B) A highly luminous dual‐site fluorescent probe, Au‐ZIF@carbon‐dots (CDs), was created by encapsulating CDs within Au‐ZIF, utilizing the inner cavity of Au‐ZIF. This serves as a foundation for developing a ratiometric fluorescence platform designed for triazophos detection. C) Integrating this ratiometric fluorescence detection platform with hydrogel and image processing algorithms enables convenient, precise, and highly sensitive detection of triazophos.

Zeolitic imidazolate frameworks (ZIFs) constitute a crucial class of porous materials with extensive applications in fields such as catalysis and separation.^[^ ^16^, ^17^, ^18^ ^]^ ZIF‐8, a well‐known ZIF formed from zinc ions and 2‐methylimidazolate, exhibits notable low toxicity and excellent biocompatibility.^[^ ^19^, ^20^, ^21^ ^]^ The dynamic interaction between 2‐methylimidazolate and zinc cation suggests the potential for encapsulating nanoparticles or molecules with relatively high affinities into ZIF‐8, while the permanent structural porosity of ZIF‐8 enables efficient loading of additional molecules, thus enhancing its capacity for guest objects.^[^ ^22^, ^23^, ^24^ ^]^ Specifically, encapsulating numerous AuNCs within ZIF‐8 significantly reduces their non‐radiative transitions and limits ligand vibrations and rotations, thereby improving luminescence intensity and stability to some extent.^[^ ^25^ ^]^ However, these methods require pre‐aggregation of AuNCs, with the aggregation degree being difficult to control, leading to inconsistent luminescence intensity.^[^ ^26^ ^]^ Additionally, light transmission of AuNCs in synthetic materials is often hindered by other components, resulting in relatively low luminescence efficiency.^[^ ^25^ ^]^ Furthermore, previous studies primarily emphasized the encapsulation capacity of ZIF‐8 without a comprehensive exploration of its potential from various perspectives, and only limited progress has been made in developing advanced strategies to optimize the biosensing performance of ZIF‐8. For instance, Su and collaborators demonstrated the possibility of positioning Au_25_(SG)_18_ nanoclusters both inside and outside ZIF‐8; however, their study mainly focused on the assembly approach, and the biosensing applications of ZIF‐8 with surface‐impregnated Au_25_(SG)_18_ were presented more as a conceptual perspective with minimal data.^[^ ^27^ ^]^ Consequently, there is a pressing need to develop a more advanced strategy that simultaneously expands the loading capacity and enhances the quantum yield of AuNCs in nanocomposites to improve luminescence performance. A simple and versatile strategy was developed through the impregnation of AuNCs onto ZIF‐8, yielding adjustable and significant luminescence properties. This method also preserves the high cavity loading capacity of ZIFs, enabling the construction of multifunctional composites. In this research, various Au‐ZIF architectures were synthesized through AuNCs‐triggered coordination of glutathione (GSH) with Zn, employing a structure‐adjustable approach based on the coordination‐dissociation mechanism. Furthermore, CDs were incorporated into the cavity of Au‐ZIFs, leveraging their low cost, photostability, and stable fluorescence emissions that resist photobleaching and background interference,^[^ ^28^, ^29^ ^]^ Point‐of‐care (POC) hydrogel sensors with enhanced optical performance were created by embedding Au‐ZIF@CDs into specially designed hydrogel discs. These hydrogel sensors exhibited advanced capabilities for detecting triazophos (TZP), utilizing the accumulation effect of ZIF‐8, the self‐calibration properties of CDs, and the significantly enhanced luminescence and targeted recognition of AuNCs. This work underscores the potential for developing controllable materials with superior biological functionalities and offers a framework for constructing highly sensitive multifunctional biosensors (**Figure** **1**).

## 2 Results and Discussion

### 2.1 Synthesis and Characterization of Au‐ZIF Composites

**Figure 2:**
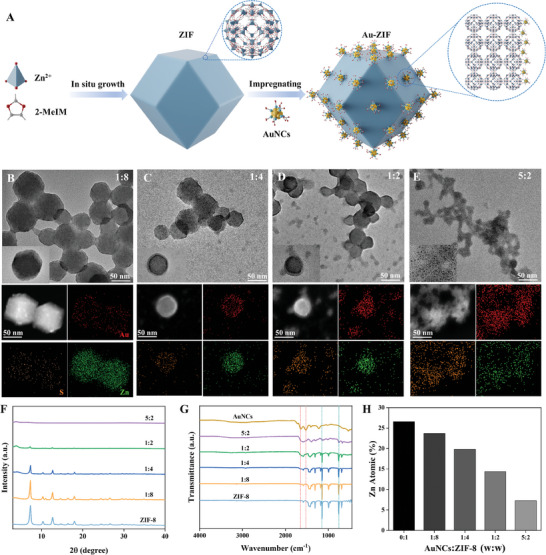
A) Illustration of the synthesis process of Au‐ZIF. B–E) TEM images and EDS mappings of Au‐ZIF nanocomposites formed with the AuNCs/ZIF‐8 mass ratio of 1:8, 1:4, 1:2, and 5:2. Scale bar: 50 nm. F) The XRD curves, G) FTIR spectra, and H) Zn atomic content in Au‐ZIF formed with different mass ratios of AuNCs/ZIF‐8.

Given the larger size of AuNCs compared to the pore size of ZIF‐8 (Figures S1–3, Supporting Information), along with the electrostatic attraction between AuNCs and ZIF‐8 (Figure S4, Supporting Information) and the ligand interactions between the carboxyl group of glutathione in AuNCs and the zinc ions in ZIF‐8,^[^ ^30^, ^31^ ^]^ AuNCs can be effectively impregnated onto the ZIF surface to form an Au‐ZIF nanocomposite (**Figure** **2**A; Figure S5, Supporting Information). An unexpected phenomenon emerged during Au‐ZIF preparation, wherein the mass ratio of AuNCs to ZIF‐8 influenced the kinetic growth of nanomaterials, favoring specific morphologies. Transmission electron microscopy (TEM) facilitates the systematic observation of the morphological evolution of Au‐ZIF, guided by AuNCs, as the mass ratio of AuNCs:ZIF‐8 increases from 1:8 to 5:2 (Figure 2B–E). The rhombic dodecahedral shape of ZIF‐8 remained unaltered, with AuNCs aggregating around ZIF‐8 at a low mass ratio of AuNCs/ZIF‐8 (1:8). However, with an increased proportion of AuNCs relative to ZIF‐8, the rhombic dodecahedral structure of ZIF‐8 transformed into a spherical shape or dissolved entirely, accompanied by exfoliated AuNCs agglomerating with Zn to form free‐floating material in the solution. As the mass ratio of AuNCs to ZIF‐8 continued to rise, the ZIF‐8 structure was progressively etched into a spherical form or completely disintegrated. Concurrently, zinc, which was initially integrated into the ZIF‐8 structure, aggregated with AuNCs, resulting in a distinct, free‐floating aggregate in the solution. The energy‐dispersive spectroscopy (EDS) elemental mappings characterized the distribution of C, Zn, S, and Au elements within the Au‐ZIF composites. Notably, the Zn and Au element distributions outside the composite confirmed the exfoliation of Zn and the aggregation of AuNCs facilitated by Zn. X‐ray diffractometry (XRD) demonstrated that the morphological differences between Au‐ZIF and pure ZIF‐8 intensified as the AuNCs/ZIF ratio increased, indicating the corrosion of the rhombic dodecahedral structure by AuNCs (Figure 2F). Fourier‐transform infrared (FTIR) spectroscopy confirmed the presence of typical AuNCs and ZIF‐8 stretching vibrations, consistent with the formation of a heterointerface (Figure 2G). Additionally, inductively coupled plasma optical emission spectroscopy (ICP‐OES) demonstrated that a higher AuNCs concentration led to a decrease in Zn mass fraction (from 26.58% to 7.28%), verifying the decomposition of ZIF (Figure 2H).

**Figure 3:**
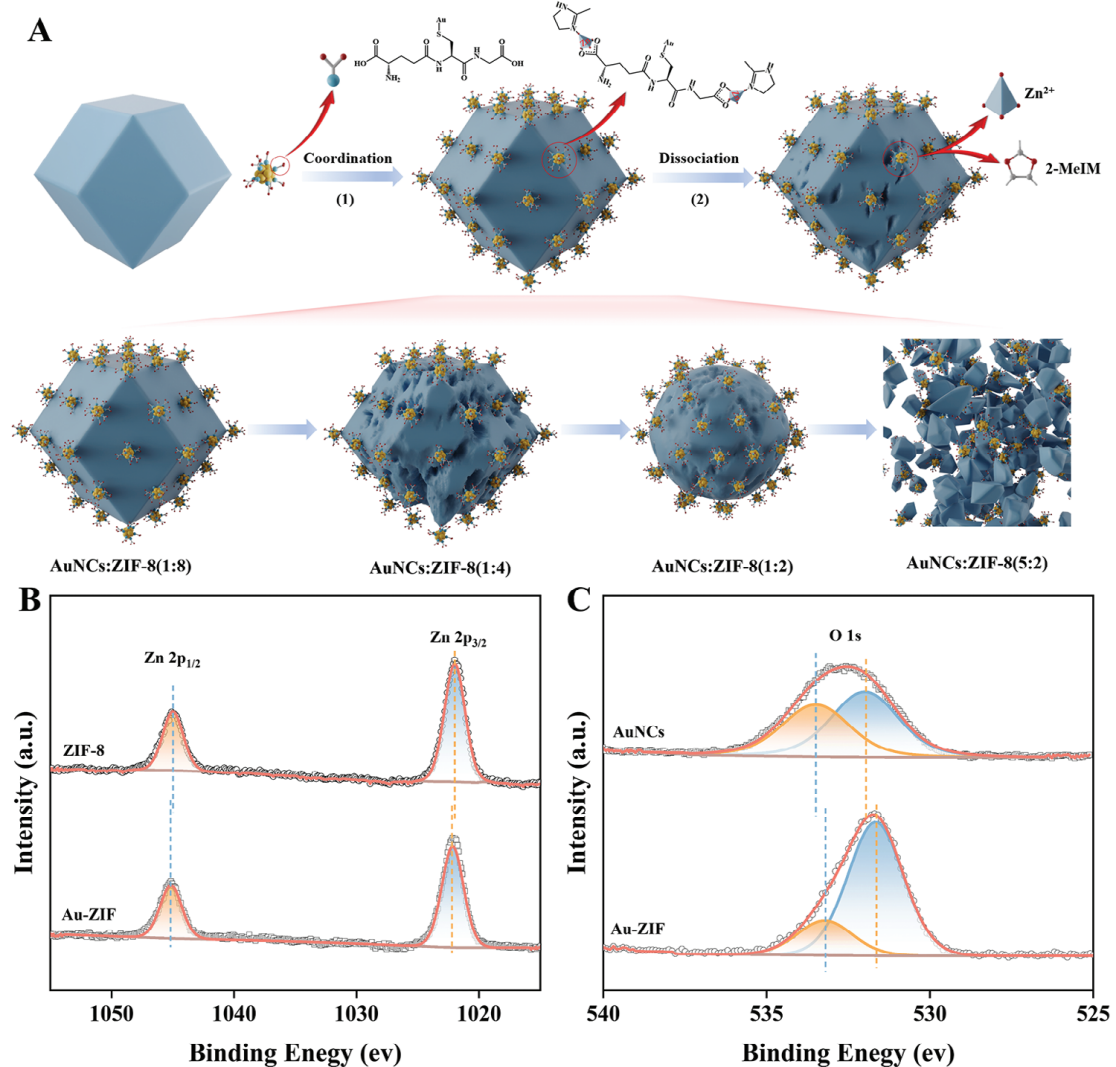
A) Coordination‐dissociation mechanism for the formation of the Au‐ZIF nanocomposites. B) Zn 2p and C) O 1s XPS spectra of ZIF‐8 and Au‐ZIF, respectively.

To elucidate the causes behind the observed changes in Au‐ZIF morphology, a comparative analysis of TEM images between Au@ZIF and Au‐ZIF was conducted. This comparison revealed a significant difference in the loading modes of AuNCs and ZIF‐8 across the two assembly strategies (Figure 2B–E; Figure S6, Supporting Information). Specifically, in the Au@ZIF assembly, AuNCs were integrated within the interior of ZIF‐8, whereas in the Au‐ZIF assembly, AuNCs were attached to the exterior surface of ZIF‐8. It is therefore proposed that the morphology of Au‐ZIF composites is precisely regulated by the externally loaded AuNCs on ZIF‐8. The underlying phenomenon is hypothesized to stem from the “coordination‐dissociation mechanism” of the Au‐ZIF assembly, involving AuNCs/Zn^2+^ coordination and subsequent ZIF dissociation (**Figure** **3**A). The carboxyl group of glutathione (GSH) in AuNCs coordinates with Zn ions, forming GSH‐Zn on the ZIF‐8 surface, which breaks the Zn‐N bonds and etches ZIF‐8. At a high mass ratio of AuNCs to ZIF‐8, numerous AuNCs coordinate with Zn, breaking multiple Zn─N bonds and leading to the collapse of the ZIF‐8 structure. In contrast, a lower AuNCs/ZIF‐8 ratio results in fewer Zn─N bonds being disrupted, thus preserving the ZIF‐8 structure. X‐ray photoelectron spectroscopy (XPS) data show that the Zn 2p peak of Au‐ZIF has a higher binding energy than that of ZIF‐8 (Figure 3B) while the O 1s peak of Au‐ZIF has a lower binding energy compared to AuNCs (Figure 3C). This is attributed to the reduced electron density on oxygen atoms and the interaction force between AuNCs and Zn, confirming the formation of Zn─O bonds and supporting the proposed hypothesis.

**Figure 4:**
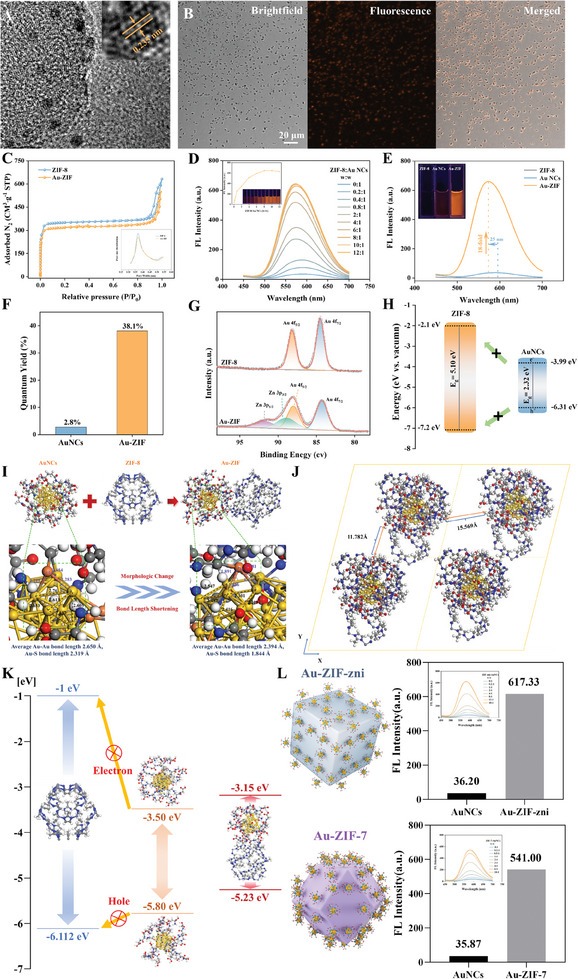
A) HRTEM image and B) Confocal fluorescence microscopy image of Au‐ZIF. C) N_2_ adsorption‐desorption isotherms and (inset) pore size distributions of ZIF‐8 and Au‐ZIF. D) Fluorescence emission spectra of Au‐ZIF at various weight ratios of ZIF‐8 to AuNCs. Inset: corresponding intensity and digital photos of Au‐ZIF under UV irradiation. E) Fluorescence emission spectra of ZIF‐8, AuNCs, and Au‐ZIF. Inset: corresponding digital photos under UV irradiation. F) Quantum yield of AuNCs and Au‐ZIF. G) High‐resolution Au 4f XPS spectra of ZIF‐8 and Au‐ZIF. H) Band edge positions of ZIF‐8 and AuNCs. I) Optimized geometry of pristine AuNCs, ZIF‐8, and the assembled Au‐ZIF structure model. J) Outside view of the optimized geometry for the assembled Au‐ZIF structure model. K) Energy alignment of the molecular orbitals of ZIF (left), AuNCs (middle), and Au‐ZIF (right). L) Fluorescence emission intensity of AuNCs, Au‐ZIF‐zni, and ZIF‐7. Inset: fluorescence emission spectra of Au‐ZIF‐zni at various weight ratios of ZIF‐zni to AuNCs and Au‐ZIF‐7 at various weight ratios of ZIF‐7 to AuNCs.

Furthermore, structurally intact Au‐ZIF (AuNCs/ZIF‐8:1/8) was selected to verify the impregnation of AuNCs onto the ZIF‐8 surface. High‐resolution transmission electron microscopy (HRTEM) confirmed that AuNCs were assembled on the ZIF‐8 surface (**Figure** **4**A). Additional evidence of AuNCs impregnation was provided by confocal laser scanning micrographs (CLSM) (Figure 4B). The Brunauer‐Emmett‐Teller (BET) assays indicated a slight decrease in the surface area of Au‐ZIF composites (1145.5 m^2^ g⁻^1^) compared to pure ZIF‐8 (1424.3 m^2^ g⁻^1^), attributed to the incorporation of nonporous AuNCs into the nanocomposites. However, the pore size distributions of ZIF‐8 and Au‐ZIF remained largely unchanged (Figure 4C). The fluorescence properties of the nanocomposites were then extensively analyzed. Notably, the fluorescence intensity of Au‐ZIF nanoparticles increased as the ratio of AuNCs to ZIF‐8 decreased, with the intensity reaching a plateau when the ratio was 1:8 (Figure 4D). This phenomenon is mainly attributed to the fact that when the ratio is reduced to a specific threshold, an optimal equilibrium is established between the structural integrity of ZIF and the loading of AuNCs. This equilibrium facilitates the optimal incorporation of AuNCs, thereby maximizing fluorescence intensity. Additionally, the supernatant of the centrifuged Au‐ZIF solution remained nonfluorescent, confirming the complete payload of AuNCs (Figure S8, Supporting Information). Consequently, Au‐ZIF (AuNCs/ZIF‐8: 1/8) was selected for further fluorescence performance analysis. Remarkably, the fluorescence emission of Au‐ZIF increased 18‐fold with a blue shift of 25 nm (Figure 4E). Moreover, AuNCs loaded onto the ZIF surface exhibited a significantly higher quantum yield, rising from 2.80% to 38.10% (Figure 4F; Figure S9, Supporting Information). The CIE chromaticity diagrams of AuNCs (0.4447, 0.4217) and Au‐ZIF (0.4787, 0.4994) further confirmed the change in fluorescence color following assembly (Figure S10, Supporting Information). Additionally, the photostability of Au‐ZIF nanocomposites was evaluated under varying solvent conditions, salt concentrations, temperatures, and photoexcitation times (Figures S11–S14, Supporting Information). The fluorescence intensity of Au‐ZIF nanocomposites displayed minimal fluctuations, whereas AuNCs alone exhibited considerable variations, indicating that the assembly strategy provided the nanocomposites with remarkable stability.

To identify the underlying mechanism of the fluorescence enhancement, the following experimental investigations were conducted. The absorption band edge of ZIF‐8 was found to occur at 344 nm. Since the excitation wavelength is 360 nm, it can be concluded that this wavelength does not produce photoinduced electrons and holes in ZIF‐8 (Figure S15, Supporting Information). Thus, the hypothesis that the fluorescence enhancement results from photon transfer from ZIF‐8 to AuNCs is dismissed. XPS analysis showed that the ratio of Au(0)/Au(I) remained nearly unchanged before and after assembly, indicating that the fluorescence enhancement of Au‐ZIF is not linked to changes in the charge state of Au (Figure 4G). After ruling out the two prior possibilities, the surface‐confinement effect of ZIF‐8 on AuNCs remains a plausible explanation. The principles of fluorescence enhancement and lifetime suggest that electrons and holes confined by a matrix with a wide band gap may facilitate recombination, leading to radiation transitions and enhanced fluorescence performance.^[^ ^32^, ^33^ ^]^ Moreover, the confinement effect of ZIF‐8 on AuNCs was verified by band edge positioning and XPS valence band (VB) assays. The calculated conduction band (CB) and VB of ZIF‐8 were −2.10 and −7.20 eV, respectively. The energies of the lowest unoccupied molecular orbital (LUMO) and the highest occupied molecular orbital (HOMO) of AuNCs were determined to be −3.99 and −6.31 eV (vs vacuum). Consequently, the excited electrons and holes in AuNCs could not transfer to the CB or the VB maximum of ZIF‐8, indicating that the microenvironment of ZIF‐8 confined the photoinduced electrons and holes of AuNCs, thereby enhancing their fluorescence performance (Figure 4H; Figures S16 and S17, Supporting Information). Furthermore, fluorescence lifetime studies were conducted to investigate fluorescence enhancement. The average fluorescence lifetime of Au‐ZIF (9.34 µs) was significantly longer than that of free AuNCs (2.93 µs; Figure S18, Supporting Information). This difference may result from the coordination bonds between ZIF‐8 and AuNCs, which restrict the intramolecular movement of AuNCs, suppressing the non‐radiative relaxation pathway and enhancing the fluorescence properties of the AuNCs. The fluorescence spectra of Au‐ZIF with varying mass ratios further supported the fluorescence enhancement mechanism. The fluorescence intensity of the Au‐ZIF nanocomposites showed a gradual increase as the proportion of AuNCs decreased, plateauing at a mass ratio of AuNCs to ZIF‐8 of 1:8 (Figure 4C). The explanation is that at higher mass ratios of AuNCs/ZIF‐8, the AuNCs surround the structurally collapsed ZIF‐8 (Figure 2B), thereby limiting the intramolecular movement of AuNCs, resulting in a minor enhancement of fluorescence. A decrease in the mass ratio of AuNCs/ZIF‐8 preserves the ZIF‐8 structure and promotes the emergence of a confinement effect (Figure 2E), which, combined with the restriction of intramolecular motions, achieves a dual enhancement of AuNCs fluorescence, significantly improving fluorescence performance.

The modifications in the electronic structure and geometrical configuration of AuNCs following their deposition onto the ZIF‐8 surface are crucial for their fluorescence properties. Therefore, the influence of the ZIF‐8 structure on the AuNCs was examined using density functional theory (DFT) calculations. Electrostatic interactions and ligand bonding between AuNCs and ZIF‐8 facilitate the rearrangement of the ligands on AuNCs, with most carboxyl groups in the glutathione ligand (SG) becoming attached to ZIF‐8. It is significant that as the ligand expands, the lengths of the Au‐Au bonds (from 2.650 to 2.394 Å) and Au‐S bonds (from 2.319 to 1.844 Å) in AuNCs gradually decrease, resulting in a pseudo‐spherical structure (Figure 4I). This change in configuration leads to a reduction in the diameter (1.71 nm) of the AuNCs on the ZIF‐8 surface, consistent with the TEM findings (1.75 nm). The DFT calculations indicate that the AuNCs are securely positioned within the microenvironment, maintained by host‐guest coordination bonding and electrostatic interactions. Simultaneously, the vibrations and rotations of the AuNCs are largely restricted, resulting in a significant decrease in non‐radiative processes. The observed enhanced fluorescence emission aligns with the findings of the experimental studies. As illustrated in Figure 4J, the nearest interparticle distance between adjacent AuNCs is 11.782 Å, surpassing the distance threshold for aurophilic interactions (3.3 Å), as reported previously.^[^ ^34^, ^35^ ^]^ This suggests that neighboring AuNCs on the ZIF‐8 surface in this study are likely to remain largely independent, with limited interactions between them. The band edge positions of ZIF‐8 and AuNCs calculated by DFT correspond to the experimental results. Furthermore, following confinement by ZIF‐8, the HOMO and LUMO of the AuNCs changed from –5.80 eV and –3.50 eV to –5.23 eV and –3.15 eV, respectively, confirming that the electronic structures of the AuNCs were affected by the external microenvironment of ZIF‐8 (Figure 4K; Supporting Information). These findings indicate that the confinement effect of ZIF‐8 on the configuration and electronic properties of AuNCs enhances fluorescent emission.

Concurrently, the extensive adaptability of this assembly strategy was examined. The versatility of the scheme was subsequently validated using different ZIFs. Based on the proposed methodology, AuNCs‐ZIF‐zni and AuNCs‐ZIF‐7 were synthesized by assembling GSH‐AuNCs with ZIF‐zni and ZIF‐7, leading to fluorescence intensity enhancements of 17.05‐fold and 15.08‐fold, respectively (Figure 4L). Thus, the ZIF‐engineered AuNCs demonstrated a significant improvement in luminescence performance through the confinement effect, suggesting the potential for this approach to be applied to other systems.

### 2.2 Fabrication of High‐Performance Au‐ZIF@CDs‐Based Biosensors

**Figure 5:**
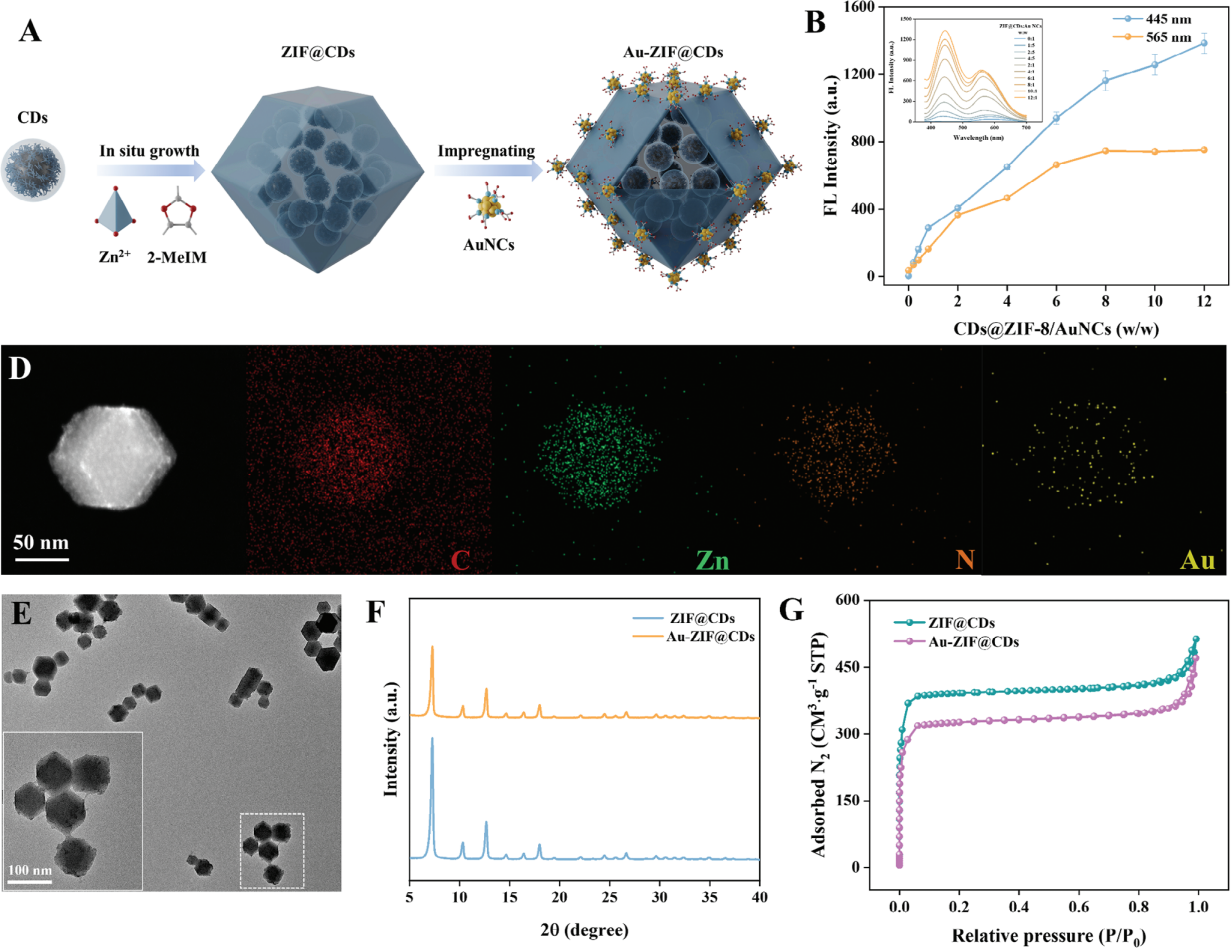
A) Illustration of the synthesis process of Au‐ZIF@CDs. B) Fluorescence emission spectra of Au‐ZIF@CDs at various weight ratios of ZIF@CDs to AuNCs. C) EDS mapping and D) TEM images of Au‐ZIF@CDs. Inset: magnified TEM images of Au‐ZIF@CDs. E) XRD patterns and F) BET analysis of ZIF@CDs and Au‐ZIF@CDs. All data presented are means ±s.d. (n = 3 independent experiments for Figure 5B).

Based on the inner cavity structure of ZIF‐8, fluorescent CDs were selected for encapsulation to synthesize ZIF@CDs, which were subsequently assembled with AuNCs to create ratiometric Au‐ZIF@CDs nanocomposites (**Figure** **5**A). As expected, the fluorescence intensity of AuNCs in Au‐ZIF@CDs reached a plateau at a ZIF@CDs/AuNCs ratio of 8:1, resulting in a 20‐fold increase in overall intensity (Figure 5B). This finding indicates that the encapsulation of CDs does not interfere with the “coordination‐dissociation mechanism” or the fluorescence performance of Au‐ZIF. Therefore, the ZIF@CDs/AuNCs ratio of 8:1 was chosen for further validation. EDS element mapping confirmed the uniform distribution of C, Zn, N, and Au throughout the Au‐ZIF@CDs architecture (Figure 5C). TEM observations clearly showed the successful assembly of AuNCs on the ZIF@CD surface, with the rhombic dodecahedral morphology preserved (Figure 5D). Additionally, XRD measurements indicated that the crystal structure of ZIF‐8 remained unchanged despite the encapsulation of CDs and the assembly of AuNCs (Figure 5E). Moreover, the BET surface area of Au‐ZIF@CDs (925.40 m^2^ g⁻^1^) was lower compared to ZIF@CDs (1108.89 m^2^ g⁻^1^), supporting that the assembly process between ZIF‐8 and AuNCs was unaffected by CD encapsulation (Figure 5F). Finally, continuous irradiation at 360 nm for 1 h revealed negligible fluctuation in the fluorescence intensity of Au‐ZIF@CDs, demonstrating satisfactory photostability (Figure S19, Supporting Information). These findings suggest that the prepared Au‐ZIF@CDs exhibit exceptional luminescence with dual emission, making them promising for sensing applications.

**Figure 6:**
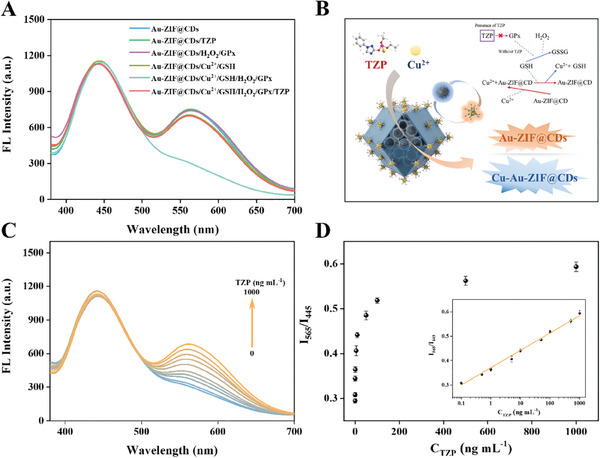
A) Feasibility of the system for the detection of TZP. B) Schematic illustrating the triazophos (TZP) detection principle. C) Fluorescence emission spectra and D) intensity ratio I_565_/I_445_ of the Au‐ZIF@CDs/Cu^2+^/GSH/H_2_O_2_/GPx system with varying TZP concentrations. The inset shows the linear relationship between the intensity ratio I_565_/I_445_ and TZP concentrations ranging from 0.1 ng mL^−1^ to 1000 ng mL^−1^. All data presented are means ±s.d. (n = 3 independent experiments for Figure 6D). Pearson's correlation coefficient was used to ascertain the linear correlation between I_565_/I_445_ and LogC_TZP_.

The Au‐ZIF@CDs nanocomposites, characterized by stable and enhanced luminescence, are highly suitable for developing sensitive and reliable biosensors with self‐calibrating capabilities. In this study, triazophos (TZP), a chemical known to pose risks to food safety, was chosen as the target analyte for detection. Due to the cation absorption properties of the zeolite‐like ZIF‐8 framework and the coordination effect of imidazole,^[^ ^36^ ^]^ Cu^2+^ ions are effectively concentrated around the Au‐ZIF@CDs nanoprobe. This enrichment leads to quenching of the orange fluorescence of AuNCs by disrupting the ligand‐Au charge transfer and facilitating electron transfer between AuNCs and Cu^2+^, showing a linear response over a broad range of Cu^2+^ concentrations (Figure S20, Supporting Information). Additionally, GSH, with stronger coordination properties compared to the carboxyl groups on AuNCs, can remove Cu^2+^ from Au‐ZIF@CDs, thereby linearly restoring the fluorescence intensity of Au‐ZIF@CDs (Figure S21, Supporting Information). Furthermore, GSH is oxidized by glutathione peroxidase (GPx) in the presence of H_2_O_2_, which linearly inhibits the aforementioned continuous reaction (Figure S21, Supporting Information). Notably, TZP, which inhibits GPx activity, releases only the GSH that coordinates with Cu^2+^, restoring the fluorescence of Au‐ZIF@CDs without affecting the blue fluorescence of CDs. This behavior highlights the potential of Au‐ZIF@CDs nanocomposites for the detection of TZP (**Figure** **6**A).

The results motivated the development of an Au‐ZIF@CDs/Cu^2+^/GSH/H_2_O_2_/GPx ratiometric assay system. The concentrations, reaction time, and pH of its components were optimized to achieve specific and reliable detection of TZP (Figure 6B; Figures S22 and S23, Supporting Information). As anticipated, a gradual increase in fluorescence was observed with increasing TZP concentrations (Figure 6C). The relationship between the fluorescence intensity ratio (I_565_/I_445_) and the logarithm of triazophos concentration (ranging from 0.1 to 1000 ng mL^−1^) fit a linear equation: I_565_/I_445_ = 0.3714 + 0.07097LogC_TZP_ (R^2^ = 0.994), with a LOD of 0.04 ng mL^−1^ (Figure 6D). Importantly, this LOD was 105 times lower than that of the AuNCs/CDs probe, indicating a substantial enhancement in sensitivity using the Au‐ZIF@CDs nanocomposite (Figure S24, Supporting Information). Additionally, the detection system exhibited high resistance to interferences from various biomolecules, pesticides, and ions, demonstrating strong anti‐interference capabilities and specificity (Figure S25, Supporting Information).

### 2.3 Evaluating the Performance of Au‐ZIF@CDs‐Decorated Hydrogels

**Figure 7:**
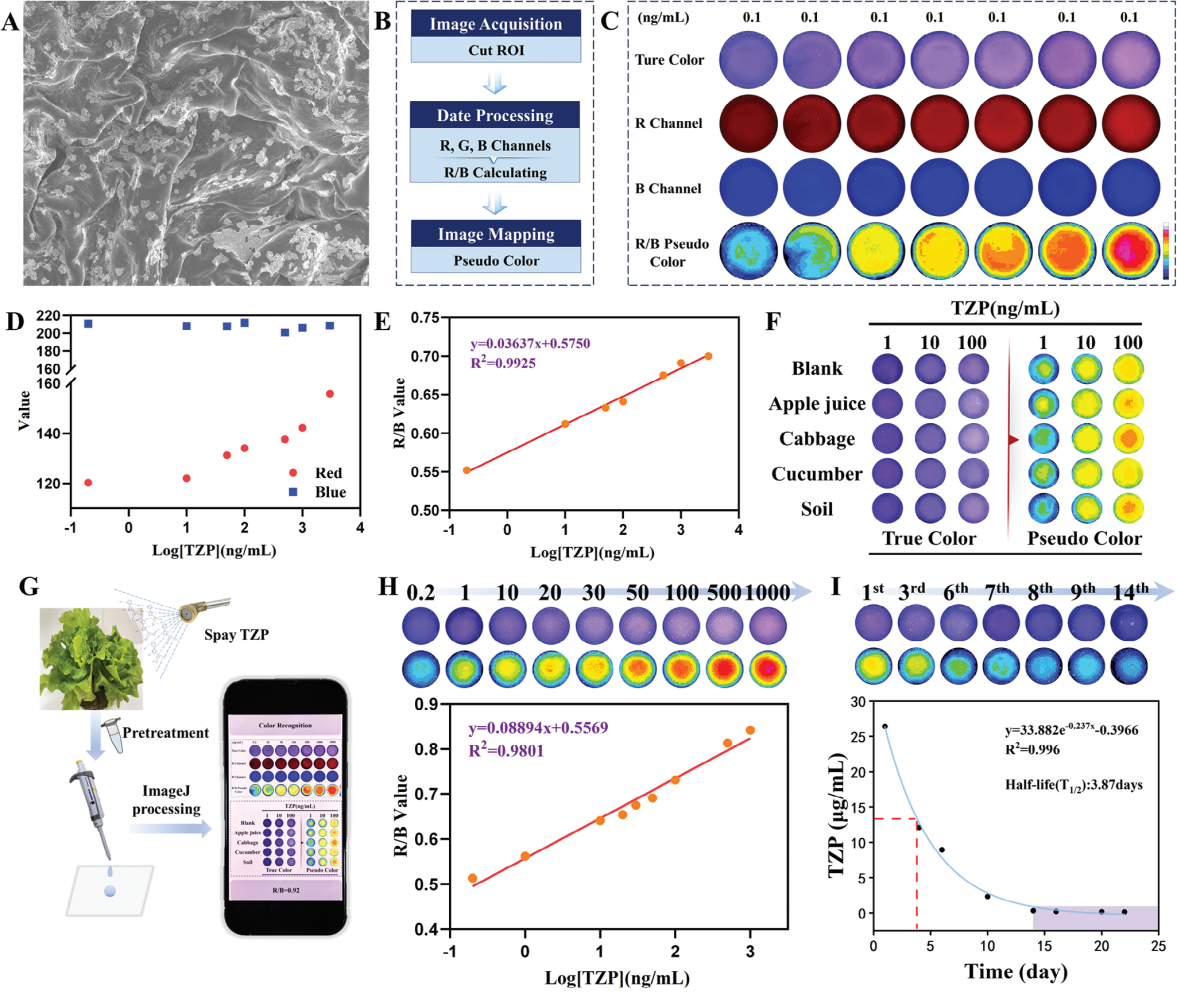
A) SEM image of the hydrogel‐based sensors. B) Image processing flow. C) R/B, R, and B values calculated from the original image. D) Relationship between R and B values and the concentration of TZP. E) Relationship between R/B response and TZP concentration. F) Raw and processed images of hydrogel‐based sensors with spiked samples. G) Operational flow of the hydrogel‐based sensors for TZP degradation. H) Relationship between the R/B value of the hydrogel‐based sensors and TZP concentration in the leaves matrix. I) TZP degradation in leaves over 22 days. Pearson's correlation coefficient was used to ascertain the linear correlation between the R/B value and Log _[TZP]_ for Panels E and H. The degradation of TZP is fitted to an exponential equation.

Hydrogel composites have found extensive application in biosensing, functioning as biocompatible and portable carriers for sensing materials.^[^ ^37^, ^38^ ^]^ In this study, a hydrogel‐based sensor was developed by integrating the Au‐ZIF@CDs composite into a hydrogel matrix using a diffusion hybridization strategy. Calcium (II)‐mediated sodium alginate junctions formed a highly porous network structure that is non‐emissive and colorless, highlighting its significant potential for bioanalytical applications.^[^ ^39^, ^40^ ^]^ The 3D, porous, and fibrous structure of the hydrogel provided a high capacity for incorporating the Au‐ZIF@CDs composite (**Figure** **7**A). Notably, the luminescence performance of the hydrogel‐based sensor remained stable despite variations in temperature, pH, and humidity, demonstrating durability over a one‐week storage period (Figure S26, Supporting Information). These findings lay the groundwork for employing Au‐ZIF@CDs‐based hydrogel sensors as robust POC devices for the detection of pesticide residues.

To POC monitoring with a clear readout signal, the fluorescence color was modulated into a transition signal. Fluorescent imaging involved maintaining a UV light source at 365 nm, along with the analyte and a fixed smartphone. Using ImageJ software, the captured true‐color image was separated into red (R), green (G), and blue (B) channels. The CDs within the complex exhibited blue fluorescence, whereas the AuNCs displayed red fluorescence. The B value remained constant as TZP concentration increased, while the R‐value increased, leading to a distinguishable channel intensity variation. The R/B intensity ratio was then calculated to amplify the sensitivity of the response signal (Figure 7B). A visual color differential map (pseudo color) was constructed by rescaling the color responses of the hydrogel‐based sensor (Figure 7C). The R/B value was utilized to quantify TZP concentration with a LOD of 0.09 ng mL^−1^, employing a linear equation (y = 0.575 + 0.03637LogC_TZP_) across a broad linear concentration range of 0.2–3000 ng mL^−1^ (Figure 7D,E). Compared to light intensity measurements, the R/B value derived from color channel analysis enhanced the sensor's accuracy. In comparison to previously reported pesticide sensors, the Au‐ZIF@CDs hydrogel‐based sensors offer significant advantages in terms of linear range, sensitivity, and analysis time (Table S2, Supporting Information). The prolonged stability and reproducibility of the hydrogel‐based sensors were evaluated by monitoring the response to TZP (100 ng mL^−1^) over 20 days (Figures S27 and S28, Supporting Information). The presence of interferents produced negligible variations in the R/B value, even at concentrations ten times higher than TZP (Figures S29 and S30, Supporting Information), demonstrating the hydrogel‐based sensors’ robust anti‐interference capability. The practical applicability and reliability of the hydrogel‐disc were further assessed by monitoring TZP in real samples. The average recoveries ranged from 91.50% to 111.62%, with relative standard deviations (RSDs) below 6.6%, indicating high accuracy for quantitative TZP determination in environmental and agricultural samples (Figure 7F; Table S3, Supporting Information). High‐performance liquid chromatography (HPLC) results further corroborated the precision and accuracy of the hydrogel‐based sensors (Figure S31, Supporting Information).

The investigation of pesticide degradation behavior in crops is crucial for minimizing pesticide exposure and improving pesticide utilization efficiency, contributing to the efficient regulation of resource inputs. The developed methods were applied to assess pesticide degradation in plants, specifically profiling triazophos (TZP) to support healthy agricultural practices. As shown in Figure 7G, TZP was sprayed onto lettuce leaves, and hydrogel‐based sensors were used to monitor the degradation over time. A fitting equation was established within the lettuce matrix to accurately track TZP degradation (Figure 7H). The efficacy of the strategy proved to be suitable for assessing the degradation of TZP residues in lettuce over a 22‐day growth period. The findings indicated that a significant amount of TZP residues (26.34 µg mL^−1^) remained in lettuce shortly after spraying. The concentration of TZP residues decreased over time, suggesting gradual degradation. The hydrogel detection method provided comparable results, with the degradation equation y = 33.882e^−0.237x^‐0.3966 and a half‐life of 3.87 days (Figure 7I). This was consistent with the results obtained from HPLC (Figure S32, Supporting Information). Monitoring throughout the 22‐day growth period revealed that lettuce reached a safe interval on the 14^th^‐day post‐application of TZP. Therefore, the proposed strategy, with its high sensitivity, stability, and accuracy, is advantageous for profiling the dynamic degradation of TZP and determining the appropriate harvest period for agricultural produce. This provides a promising tool for evaluating food safety risks associated with pesticide residues during plant growth.

## 3 Conclusion

In general, the Au‐ZIF@CDs nanoprobe has been designed to improve luminescence and stability for the sensitive detection of triazophos residues. The morphological evolution of ZIF‐8 induced by AuNCs is thoroughly examined, identifying the assembly and etching‐based mechanisms of Au‐ZIF. It was elucidated that AuNCs influence morphology through a coordination‐dissociation mechanism and were confined by the surface of ZIF‐8, restricting molecular movement and electron transfer. This hierarchically structured Au‐ZIF offers promising opportunities for ultrasensitive sensors and features internal pores for multifunctional applications. The versatility of the protocol is demonstrated by varying the types of ZIFs. Based on the structural characteristics of the aforementioned design materials, CDs were utilized as encapsulants to create Au‐ZIF@CDs nanoprobes, which displayed dual signals and were employed for TZP detection based on Cu‐bursting AuNCs. The integration of hydrogels and a smartphone‐based portable device facilitated the achievement of a detection limit at the picogram per milliliter level, significantly surpassing the sensitivity of conventional biosensors. This work not only proposes an effective strategy for the preparation of Au‐ZIF nanoarchitecture with excellent luminescence performance, but also offers multifunctionality through the encapsulation of various guests within the porosities of ZIFs. This study presents a method for demonstrating the scalable capacity of ZIF to enhance the fluorescence characteristics of AuNCs and encapsulate additional elements, enabling the creation of multifunctional composites for the sensitive identification of targets. However, the enhancement of the quantum yield of individual AuNCs and the optimization of space utilization outside the carriers remain areas requiring further investigation. Additional studies are needed to improve these properties.

## 4 Experimental Section

Experimental details are provided in the Supporting Information.

## Conflict of Interest

The authors declare no conflict of interest.

## Supporting information



Supporting Information

## Data Availability

The data that support the findings of this study are available from the corresponding author upon reasonable request.
