# Supplementary material for: Accurately Tunable AuNC‐ZIF Content Architecture Based on Coordination‐Dissociation Mechanism Enables Highly Brightness Dual‐Site Fluorescent Biosensor
Source: Adv Sci (Weinh). 2024 Dec 4;12(4):2408400. doi: 10.1002/advs.202408400 (PMC11775526; doi:10.1002/advs.202408400)
Supplement: Supplementary file 1 — Supporting Information [file ADVS-12-2408400-s001.docx]

**Structurally Tunable AuNCs−ZIF@Component Architecture Enables Highly Sensitive Dual-Site Fluorescent Biosensor**

*Junyang Chen, Yuqian Wang, Runpu Shen, Wei Li, Sainan Gao, Zhikang Xiao, Qiyan Lv, Xiaojie Song, Jianzhong Xu, Gaoxiang Xu*, Huifang Cui*, and Zhaohui Li**

**Experiment section**

**1. Material and instruments**

All of the merchant reagents with analytical grade were purchased and used without further processing required. 2-Methylimidazole(2-MIM,98%), Glutathione (GSH), Glutamate (Glu), triazophos, fomesafen, propyzamide, fenpropathrin, imidacloprid, chlorothiazide, and bifenthrin were got from Aladdin Reagent Co. Ltd (Shanghai, China). Isoproturon, atrazine, Alanine (Ala), and Histidine (His) were obtained from Shanghai Maikelin biochemical technology Co., Ltd (Shanghai, China). Glutathione Peroxidase (GPx) was obtained from Shanghai Acmec Biochemical Technology Co., Ltd (Shanghai, China). Phenylalanine (Phe), Glucose (Glc), and Fructose (Fru) were obtained from Beijing Dingguo Changsheng Biotechnology Co., Ltd. Tetrachloroauric (III) acid (HAuCl_4_) were purchased from Sangon Biotech Co., Ltd (Shanghai, China). Zinc nitrate hexahydrate (Zn(NO_3_)_2_·6H_2_O) was purchased from Fuchen Chemical Reagent Co. Ltd (Tianjin, China). We used ultrapure water with resistivity higher than 18.2 MΩ cm to prepare aqueous solutions.

The transmission electron microscopy (TEM, FEI-Tecnai G2, USA) and scanning electron microscope (SEM) were employed to observe morphological structures. Fluorescence measurements were performed on an F-7100 spectrophotometer (Hitachi, Japan). The zeta potentials were measured on Malvern Zetasizer Nano ZS90. An AXIS SUPRA XI electron spectrometer (Shimadzu, Japan) was applied to collect X-ray photoelectron spectroscopy (XPS). The UV-vis absorbance was obtained by using a U-3900H spectrometer (Shimadzu). The fluorescence lifetimes were measured with the FLS 980 fluorometer (Edinburgh, England). X-ray diffraction (XRD) analysis was used a Bruker AXS D8 Advance Powder X-ray diffractometer. FT-IR spectra were recorded with Nicolet 6700 FT-IR spectrometer (Thermo).

**2. Synthesis of AuNCs**

The GSH stabilized AuNCs were prepared based on a previously reported method^1^. Typically, fresh aqueous solution of GSH (0.8 mL, 100 mM) and HAuCl_4_ (0.3 mL, 100 mM) were added to 28.9 mL of distilled water. The mixture was stirred at room temperature for 5 min, and then stirred at 70 ℃ for 24 h. After cooling to room temperature, an equal volume of methanol was added to precipitate the AuNCs, and the suspension was centrifugated at 12000 rpm. The resulting precipitation was washed with methanol 3 times to remove the unreacted precursors. The final precipitation was re-dispersed in distilled water and stored at 4 ℃ for future use.

**3. Synthesis of CDs**

The water-soluble CDs were synthesized based on hydrothermal polymerization between citric acid and ethylenediamine^2^. In brief, citric acid (1.0507 g) and ethylenediamine (335 μL) were dissolved in distilled water (10 mL). Then the solution was transferred to a poly(tetrafluoroethylene) Teflon-lined autoclave and heated at 240 °C for 8 h. After the reaction, the reactors were cooled down to room temperature. The product was brown-black and transparent, which was further purified using dialysis bag (1000 Da molecular weight cutoff).

**4. Synthesis of ZIF@CDs**

ZIF@CDs were synthesized by adding zinc nitrate hexahydrate (149 mg) and 2-methylimidazole (328 mg) into 15 mL methanol with 400 uL CDs under stirring conditions for 24 h. The production was obtained by centrifugation at 8000 rmp for 10 min and washed with methanol for three times. Finally, the production was dissolved in distilled water (4 mg/mL) and stored at 4 ℃. Pure ZIF-8 was synthesized using a similar procedure in the absence of CDs.

**5. Synthesis of Au-ZIF and** **Au-ZIF@CDs**

Au-ZIF was prepared by mixing AuNCs (1 mg/mL, 500 μL) and ZIF-8 (4 mg/mL, 500 μL) at room temperature for 10 min under sonication conditions. The as-prepared Au-ZIF was kept at 4 ℃ for future use. Au-ZIF@CDs was also prepared by a similar process except for using ZIF@CDs replacing ZIF-8.

**6. Fluorescence Sensors for Triazophos Detection**

Various concentrations of triazophos (0, 0.1, 0.5, 1, 5, 10, 50, 100, 500, 1000 ng/mL, respectively) were incubated with GPx (10 mU/mL) for 25 min. Then, GSH (10 µM), Cu^2+^ (1.5 µM), HEPES buffer solution (pH=7.8), and Au-ZIF@CDs (40 µL) were added to the mixture and incubated for 20 min. The fluorescence spectra of the mixture were measured with excitation wavelength at 360 nm.

**7. Preparation of Hydrogel-based Sensors for Triazophos Detection.**

To make the hydrogel-based sensors, we made a master mold for casting the gel discs using a quartz slide. The master mold holds four rows of wells 10 mm in diameter and 1 mm in depth to create gel sensors. The sodium alginate (2%) and Au-ZIF@CDs mixture were loaded into a well using a pipet followed by immersing into a CaCl_2_ (0.02 M) solution for 5 min. The hydrogel discs were released from the master mold manually using forceps and stored with ultrawater.

Various concentrations of triazophos were incubated with GPx (10 mU/mL) for 25 min. Then, GSH (10 µM) and Cu^2+^ (1.5 µM) dissolved in HEPES buffer (pH=7.8) were added and incubated for 20 min. 30 μL of the reaction solution was dispensed onto the hydrogel discs. The liquid was allowed to cover, spread, and diffuse throughout the entire hydrogel discs by capillary action. After that, the fluorescence image of hydrogel discs was collected and then analyzed using the commercial software ImageJ to analyze the data information.

**8. Detection of** **Triazophos in Real Sample**

The fluorescence and hydrogel-based sensors was applied to triazophos detection in agricultural and environmental samples including apple, cabbage, cucumber, and soil. Apple, cabbage, and cucumber samples were purchased from local market and the soil was dug from local farmland. Apple samples were extracted with 5 mL of acetonitrile and 5 mL of distilled water by sonication for 30 min. The mixtures were centrifuged at 8000 rmp for 10 min and the obtained supernatant was applied to sensing assays based on above procedures. The extracted processes for cabbage, cucumber, and soil were similar to that performed for apple. Above all, real samples spiked with triazophos standards of 1, 10, and 100 μg L^-1^ were investigated for recovery study. All assays were repeated thrice.

**9. Monitoring Triazophos Degradation in Lettuce**

Two groups of lettuce were cultivated for 20 days. One group was sprayed with 20 mL of a triazophos standard solution (1.0 mg/mL) and the other was employed as a comparison. After spraying the pesticide, 2 g of lettuce were picked at 2nd, 3rd, 4th, 5th, 6th, 7th, 10th and 14^th^ days. The samples were added to 5 mL of acetonitrile and 5 mL of distilled water, then were subjected to ultrasonic treatment for 10 min. After centrifugation and filtration through a 0.22 μm organic film, the triazophos concentration in the extract solution was detected using HPLC and our hydrogel-based sensors.

**10. Detailed Calculations of the Band Edge Placement**

As for ZIF-8 and AuNCs, VB and CB of ZIF-8 and HOMO and LUMO positions of AuNCs were calculated according to the previous calculation methods^3^. The VB position (HOMO) versus normal hydrogen electrode (NHE) can be obtained by the following formula: E_VB_ (vs. NHE) = Φ + VB_max_ − 4.44, where E_VB_ (vs. NHE) is potential versus normal hydrogen electrode (NHE), Φ is the electron work function of the instrument (4.50 eV), VBmax was determined based on the XPS valence band spectra, the level of NHE is –4.44 eV with respect to the vacuum level [3]. The CB positions (LUMO) versus NHE can be obtained by the following formula: E_CB_ (vs. NHE) = E_VB_ (vs. NHE) – E_g_, where E_g_ is the band gap and determined by extrapolating a straight line through the leading edge of the spectra. As shown in Figure 3G, E_g_ values of ZIF-8 and AuNCs were determined to be 5.10 and 2.32 eV, respectively. As shown in Figure 3H, VB_max_ values of ZIF-8 and AuNCs were calculated to be 2.70 and 1.81 eV, respectively. Thus, VB and CB of ZIF-8 were calculated to be 2.76 and -2.34 V vs NHE (−7.20 eV and −2.10 eV with respect to the vacuum level). HOMO and LUMO of AuNCs were estimated to be 1.87 and −0.45 V vs NHE (−6.31 eV and −3.99 eV with respect to the vacuum level).

**11. Density Functional Theory (DFT) Calculation:**

DFT calculations were performed using Dmol3 code^4, 5^. Generalized gradient approximation functional treated by PerdewBurke-Ernzerhof was used to represent the interactions between electrons and ion cores^6, 7^. The parameter criteria of the tolerances for energy and self-consistent field convergence were 10-4 Ha and 10-5 Ha, respectively. The van der Waals correction (DFT-D) method was used by the GGA functional^8^. The smearing value was set 0.005 Ha for rapid convergence. The structure optimization was performed adopting Forcite program with COMPASSⅡ force.

**12. Statistical Analysis:**

Data were normalized at the pre-processing stage and then expressed as a mean ± standard deviation after the elaboration. The software used for initial data processing was Microsoft Excel 2021. lmaging data were acquired and processed by lmage J (National Institutes of Health, USA), respectively. The OriginPro 2021 (OriginLab, USA) and integrated functionalities were used to perform the statistical analysis and prepare the related graphs.

The limit of detection (LOD) of the sensors for TZP was calculated using the below formula:

$$\text{LOD }=\frac{3\times\text{ Standard deviation of the fitted line }}{\text{ Slope of the fitted line }}$$

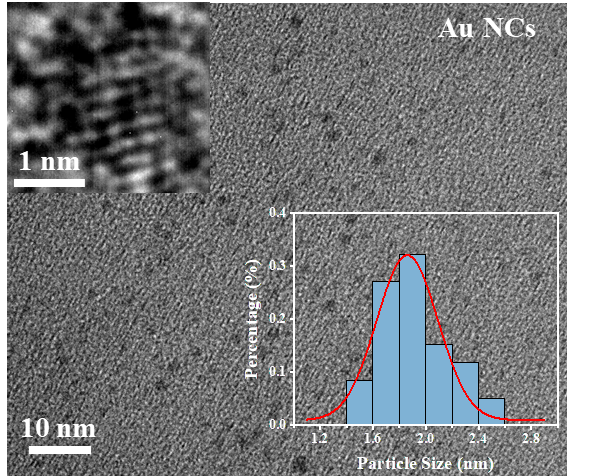


**Figure S1.** TEM images of AuNCs with the average size of 1.89 nm, inset displayed the HRTEM and size distribution of AuNCs.

**Figure S2.** The hydrodynamic diameters of AuNCs.


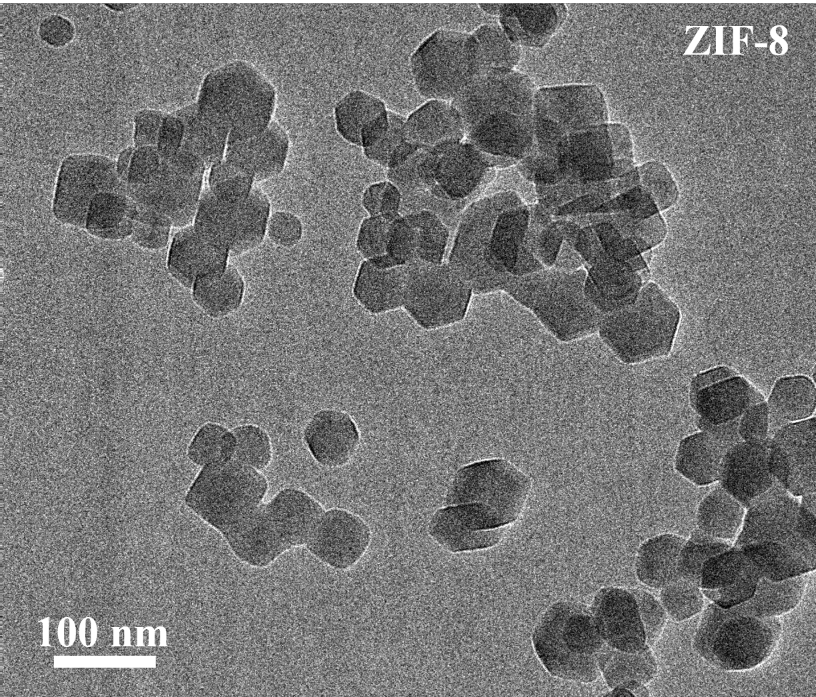


**Figure S3.** TEM images of ZIF-8.


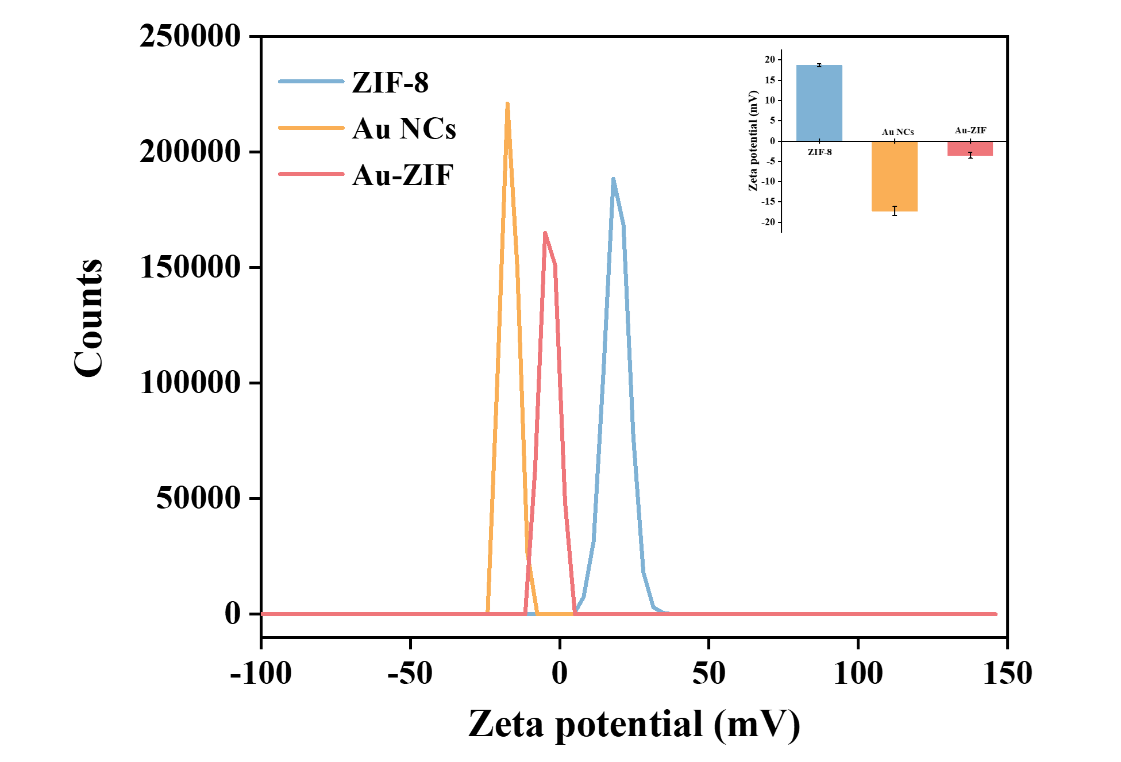


**Figure S4.** Zeta potentials of ZIF-8, AuNCs, and Au-ZIF (AuNCs/ZIF-8 = 1/8).

**
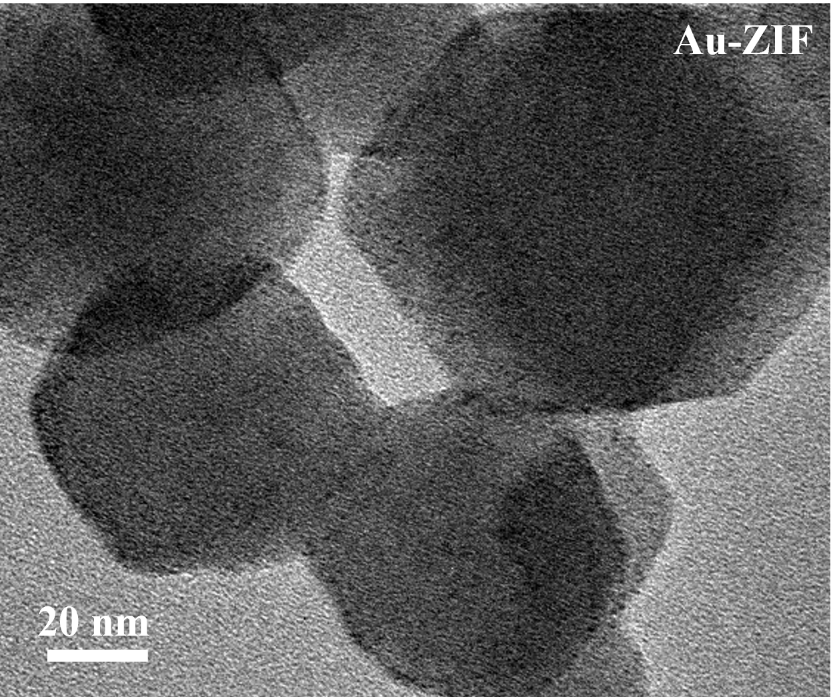
**

**Figure S5.** TEM images of Au-ZIF (AuNCs/ZIF-8 = 1/8).

**
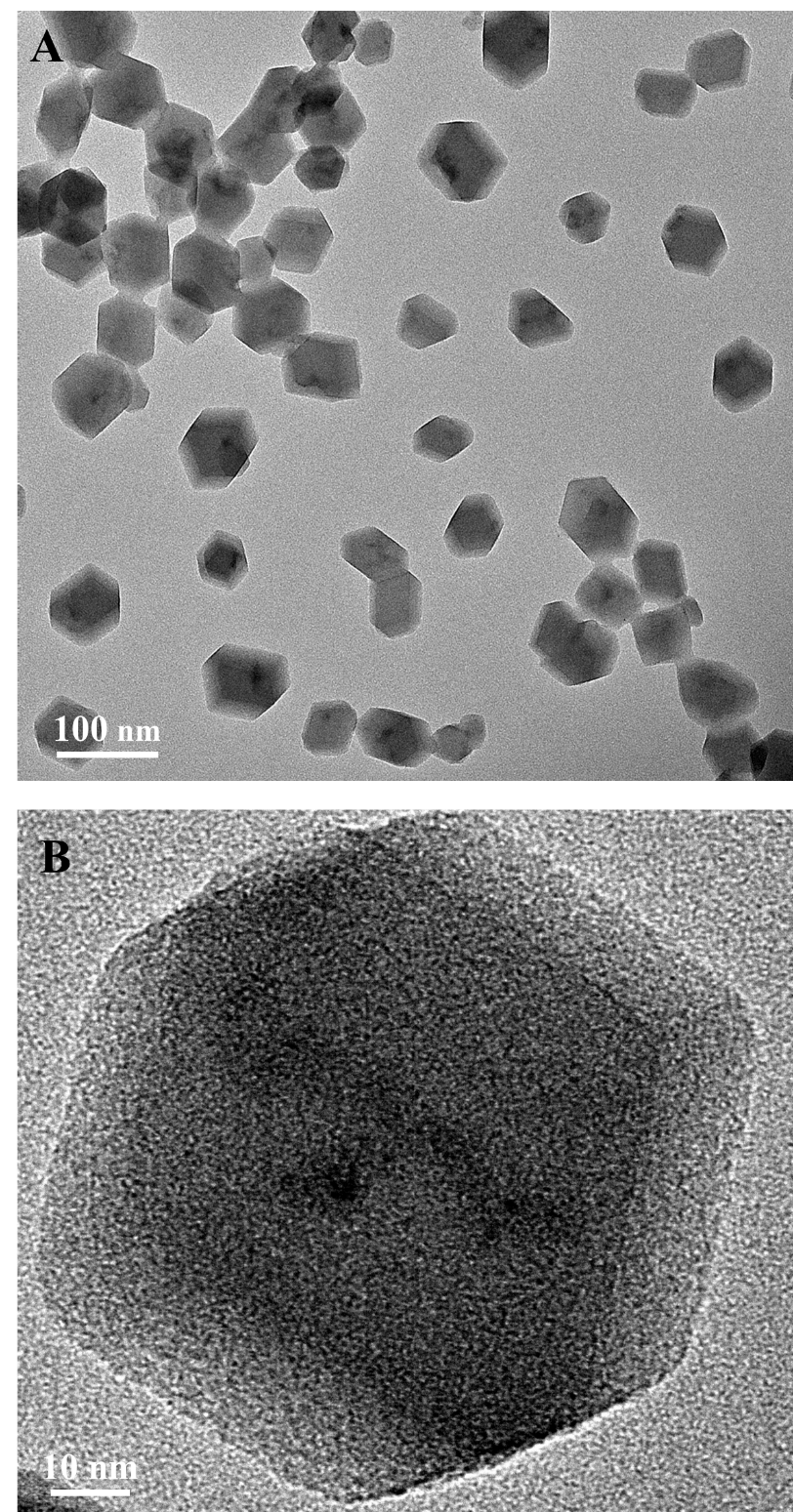
**

**Figure S6.** The TEM images of ZIF@AuNCs (AuNCs were encapsualted in ZIF-8, which was synthesized by in situ strategy).


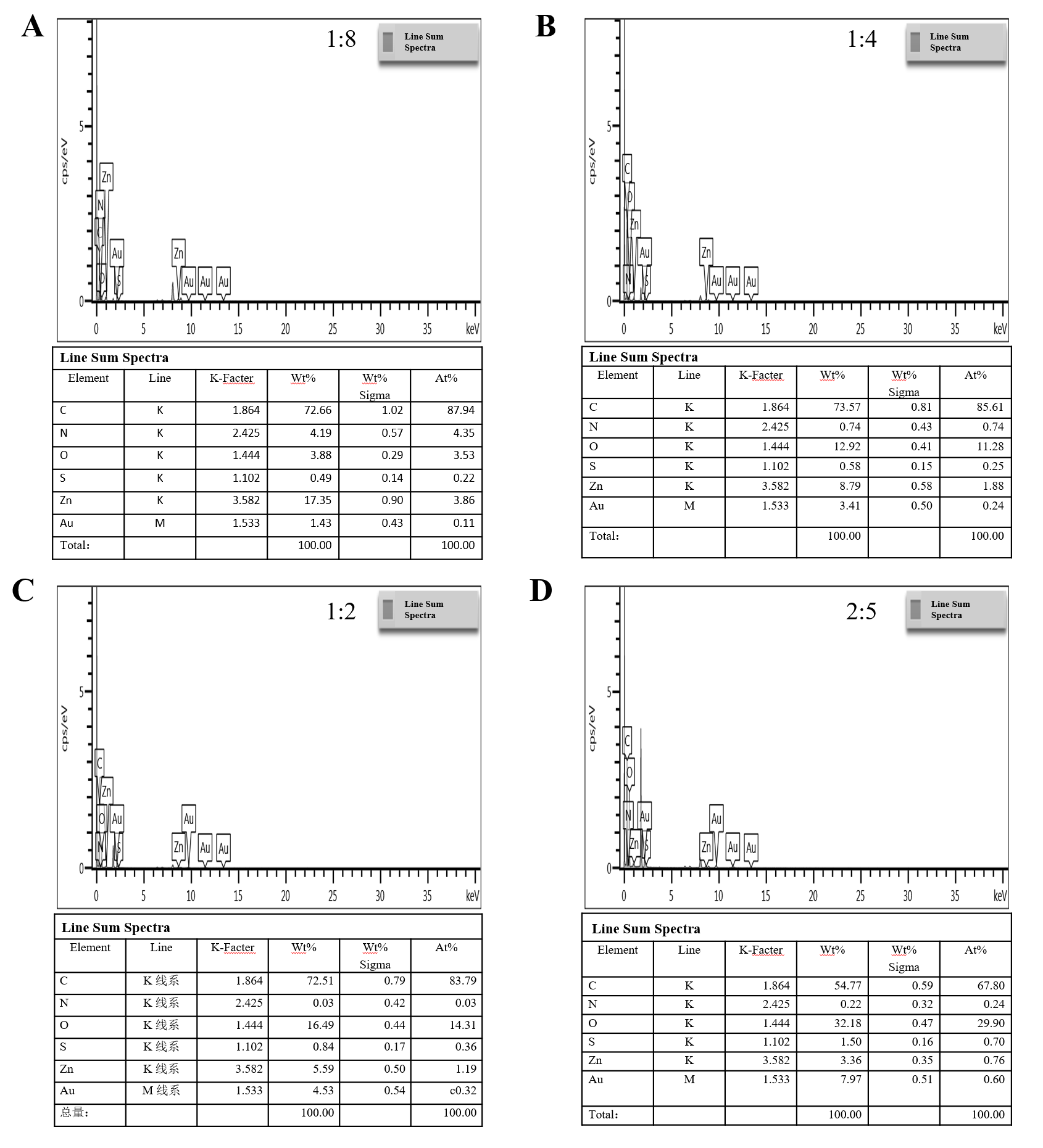


**Figure S7.** The Energy Dispersive Spectrometer (EDS) of Au-ZIF composites formed with the AuNCs/ZIF-8 mass ratio of 1:8, 1:4, 1:2, and 5:2.


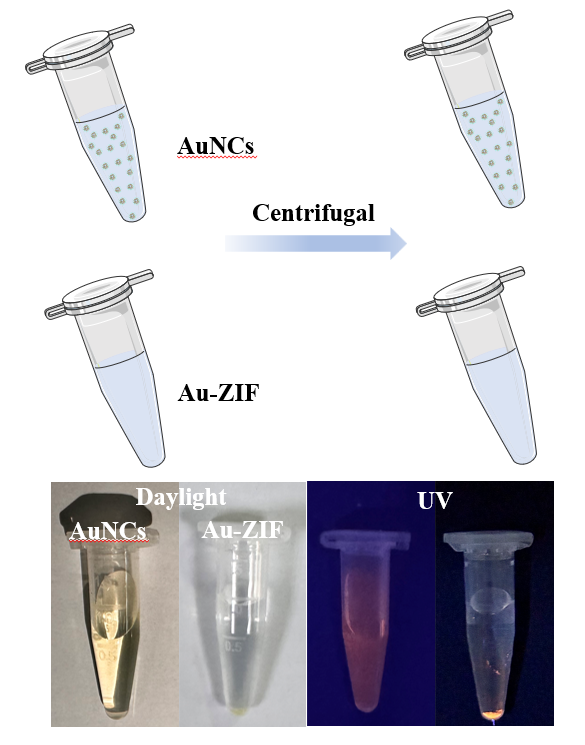


**Figure S8.** Photographs of AuNCs and the Au-ZIF-8 after centrifugation (8,000 rpm for 10 min) taken under daylight and an UV lamp, respectively.


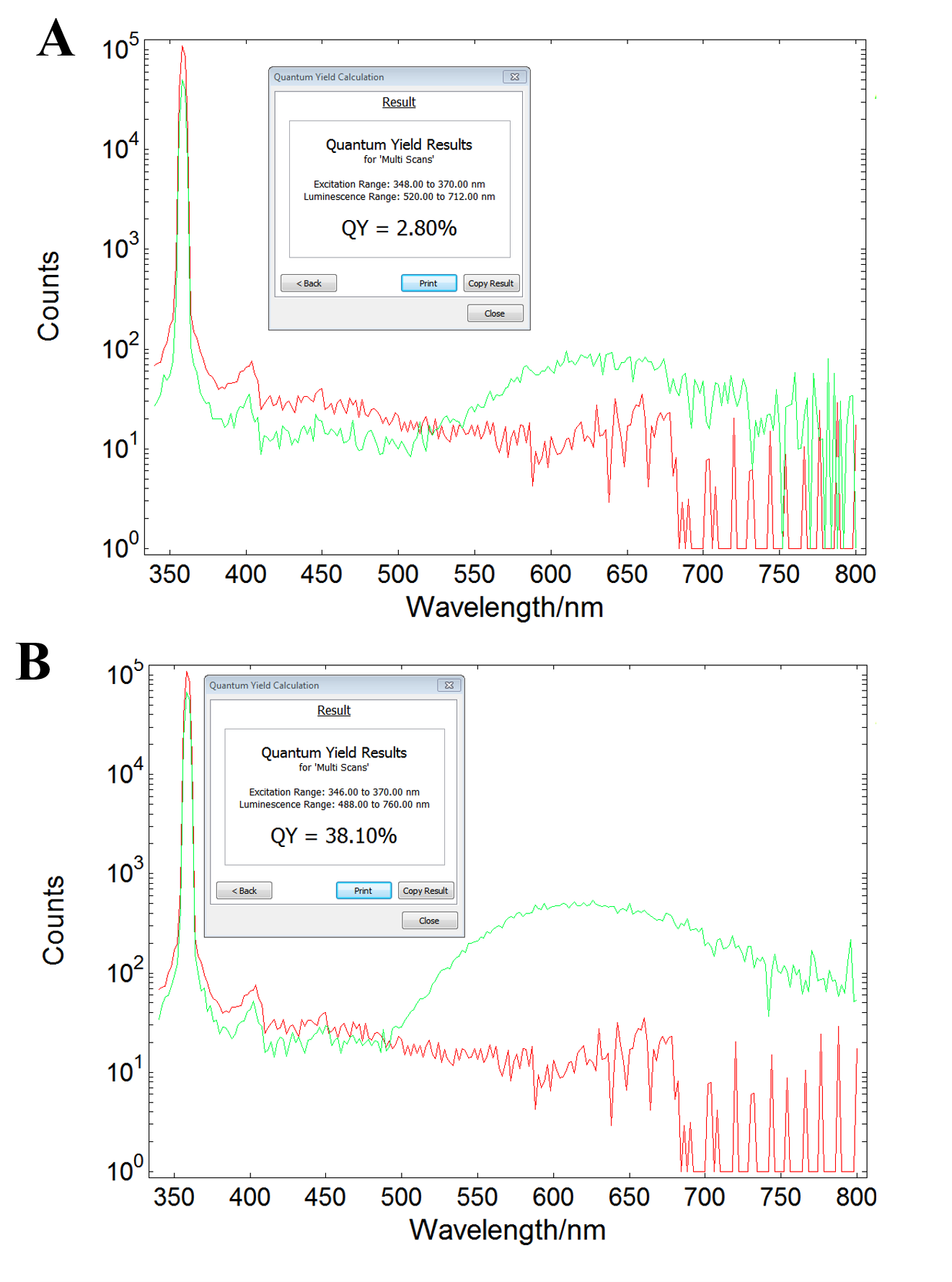


**Figure S9.** The quantum yield of (A) AuNCs and (B)Au-ZIF.

**
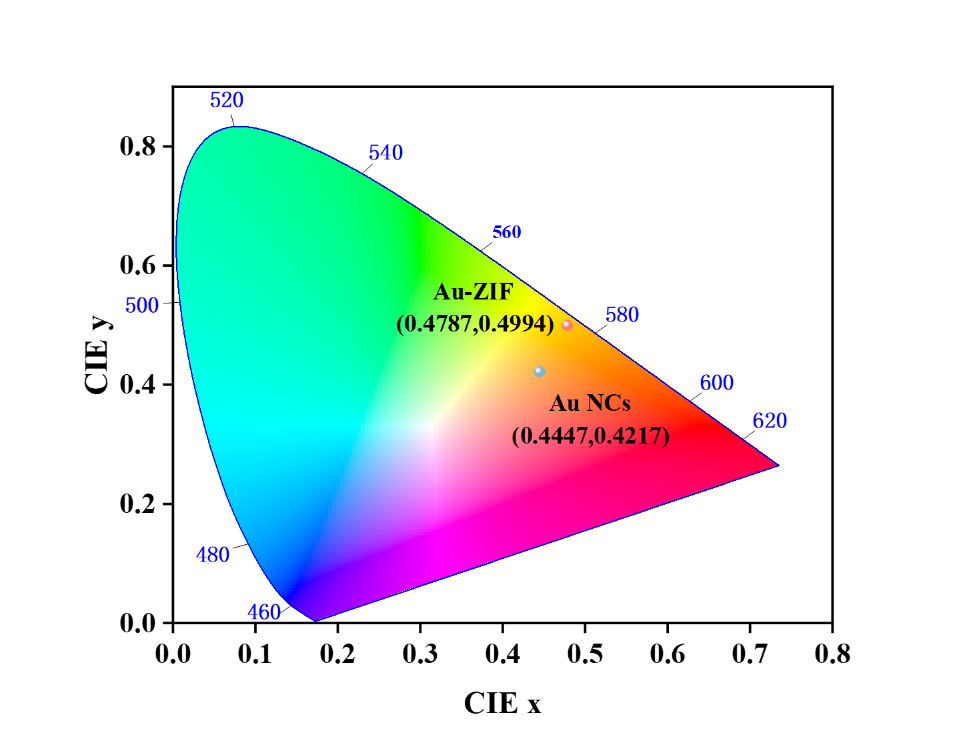
**

**Figure S10.** The stability of AuNCs and Au-ZIF in the presence of NaCl.


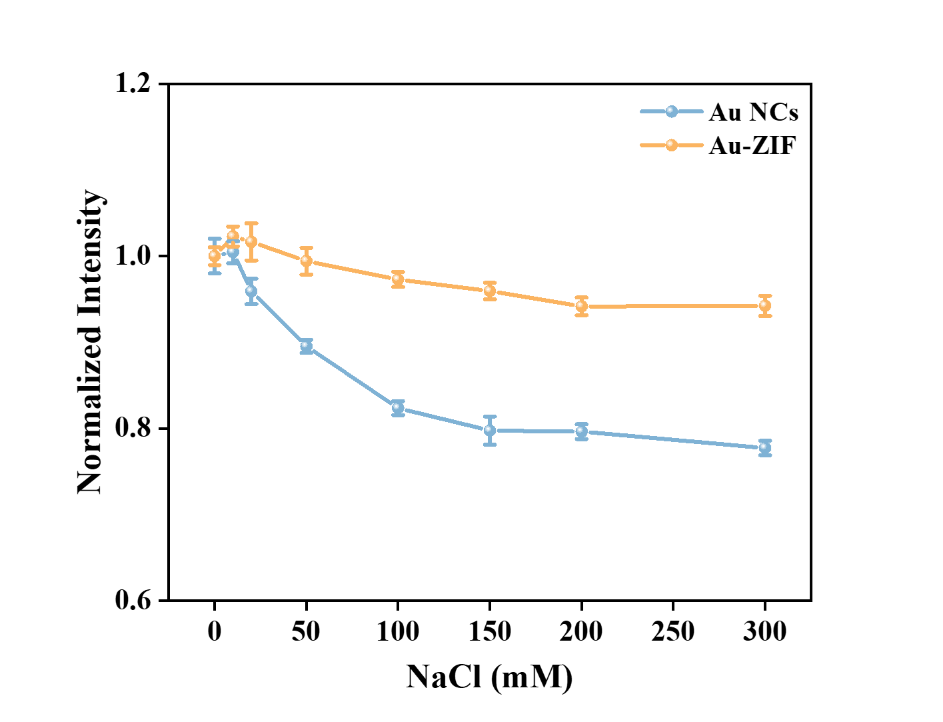


**Figure S11.** The stability of AuNCs and Au-ZIF in the presence of NaCl.


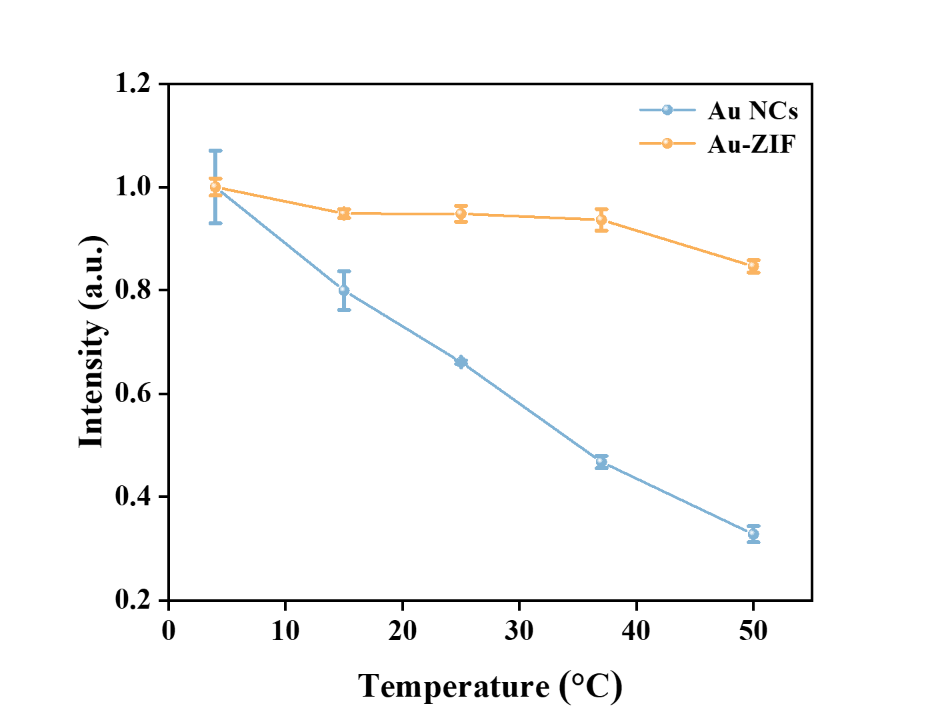


**Figure S12.** The stability of AuNCs and Au-ZIF at different temperature.


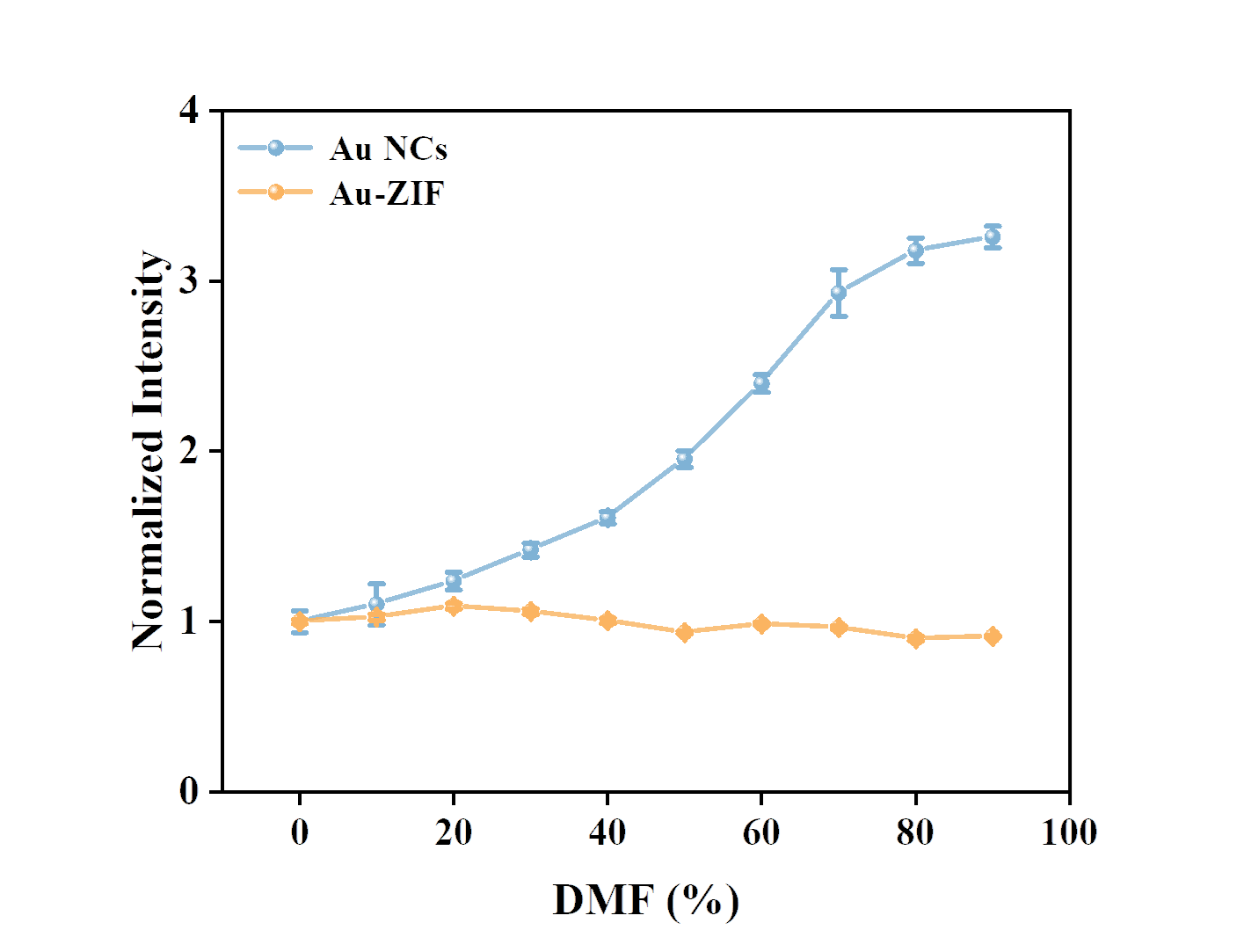


**Figure S13.** The stability of AuNCs and Au-ZIF in the presence of DMF.

**Figure S14.** The normalized fluorescence intensity of AuNCs and Au-ZIF under different radiation times of xenon lamp.


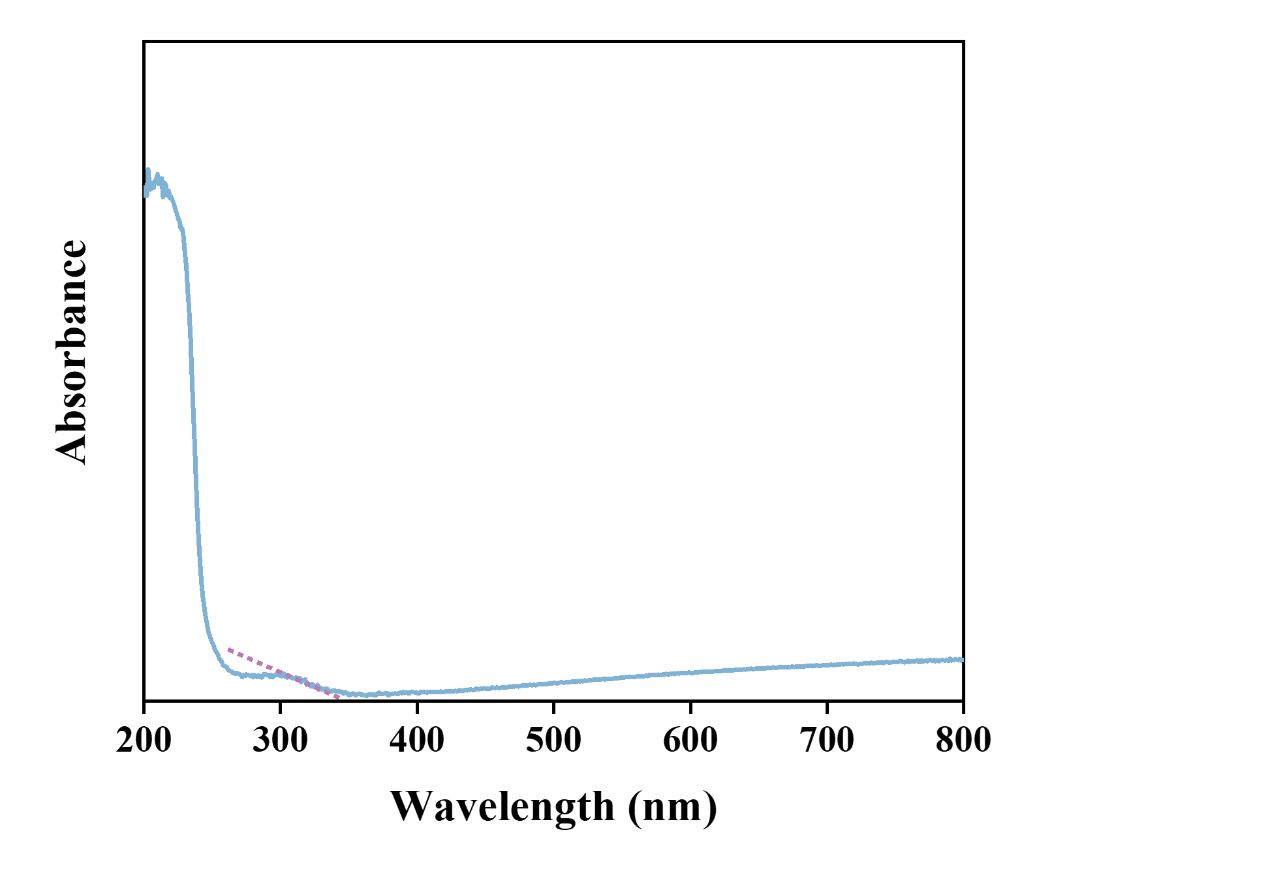


**Figure S15.** UV–vis diffuse reflectance spectroscopy of ZIF-8 solid.


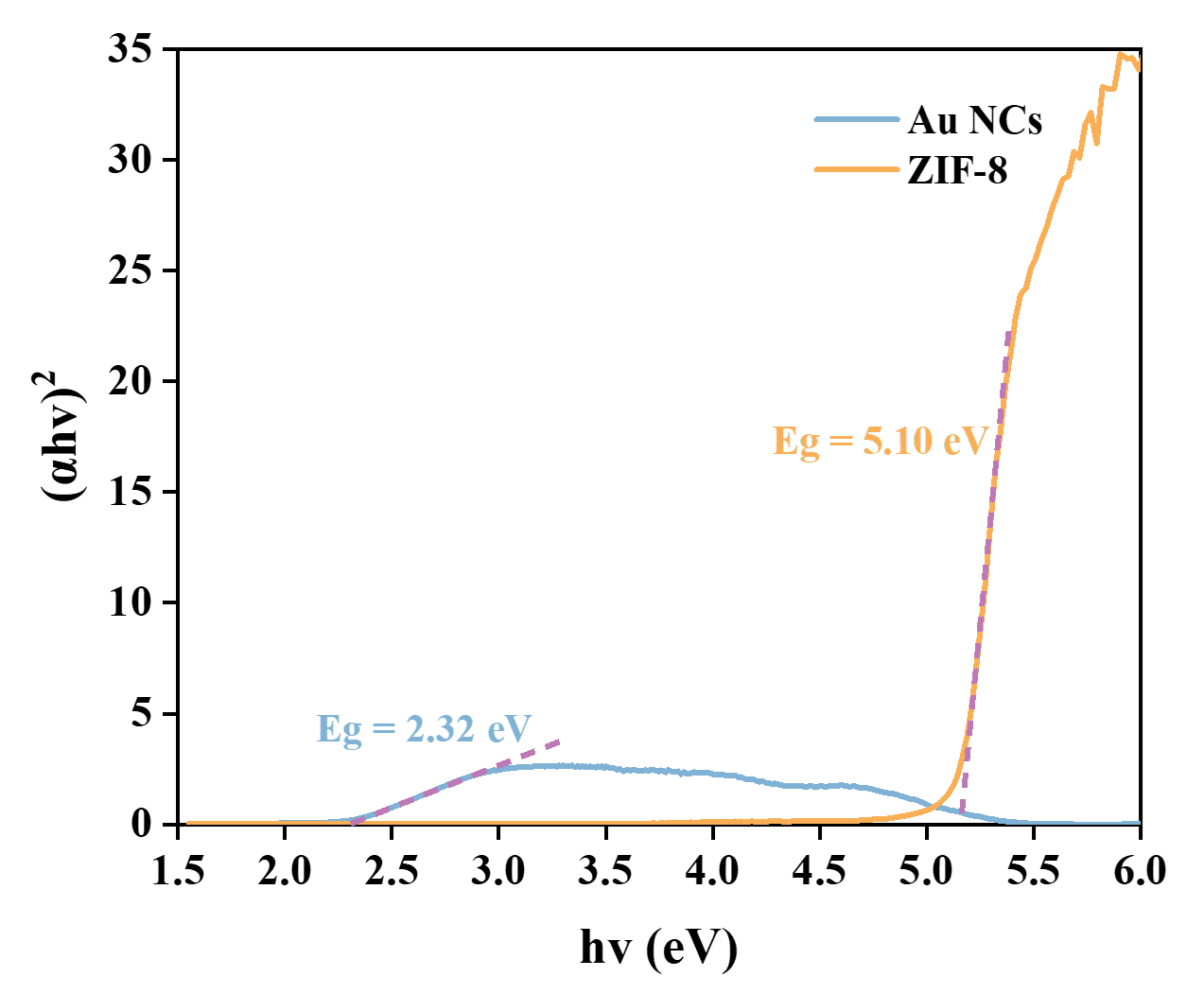


**Figure S16.** Band gap energies of AuNCs and Au-ZIF.

**Figure S17.** XPS valence band spectra of AuNCs and Au-ZIF


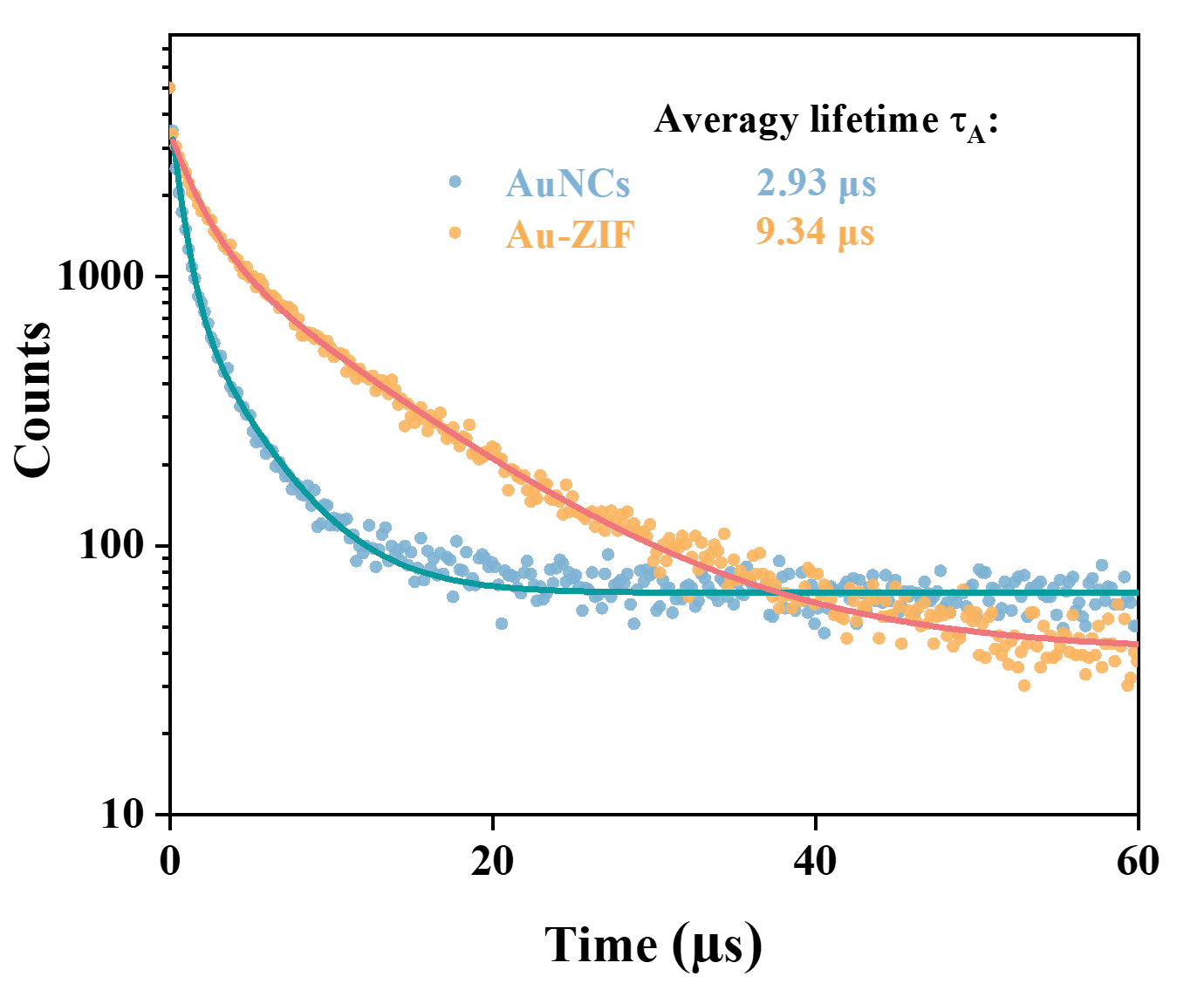


**Figure S18.** Fluorescence decay curves of AuNCs and Au-ZIF.


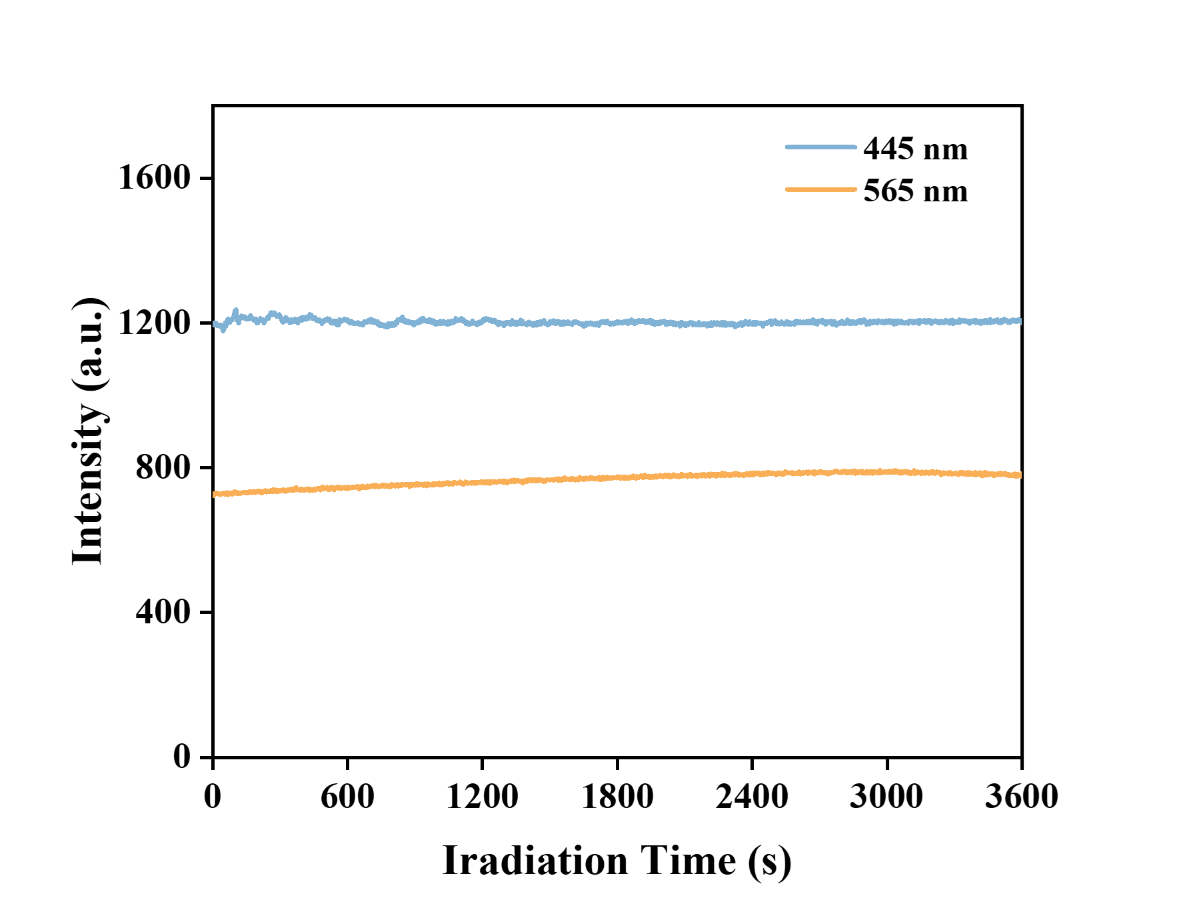


**Figure S19.** The fluorescence intensity of Au-ZIF@CDs under different radiation times of xenon lamp.


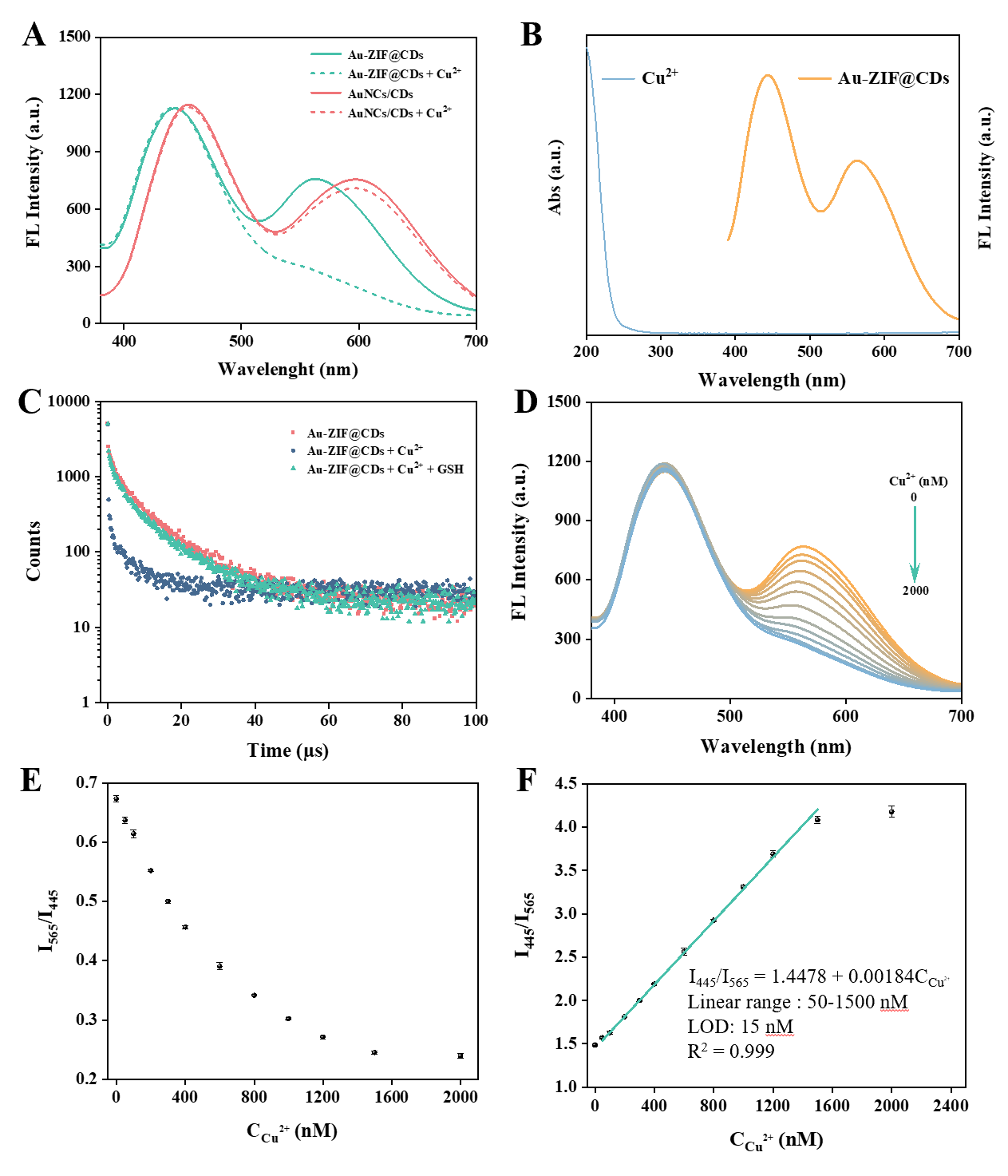


**Figure S20.** (A) Fluorescence spectra of Au-ZIF-8@CDs and AuNCs/CDs probes without/with Cu^2+^. (B) Absorbance spectrum of Cu^2+^ and fluorescence emission of Au-ZIF-8@CDs. (C) Fluorescence lifetimes of Au-ZIF-8@CDs without or with Cu2+ and Cu2++GSH. (D) Fluorescence spectra of Au-ZIF-8@CDs in the presence of various concentrations of Cu^2+^. (E) Plot of I_565_/I_445_ versus Cu^2+^ concentration. (F) The linear relationship between I_445_/I_565_ and Cu^2+^ concentration.


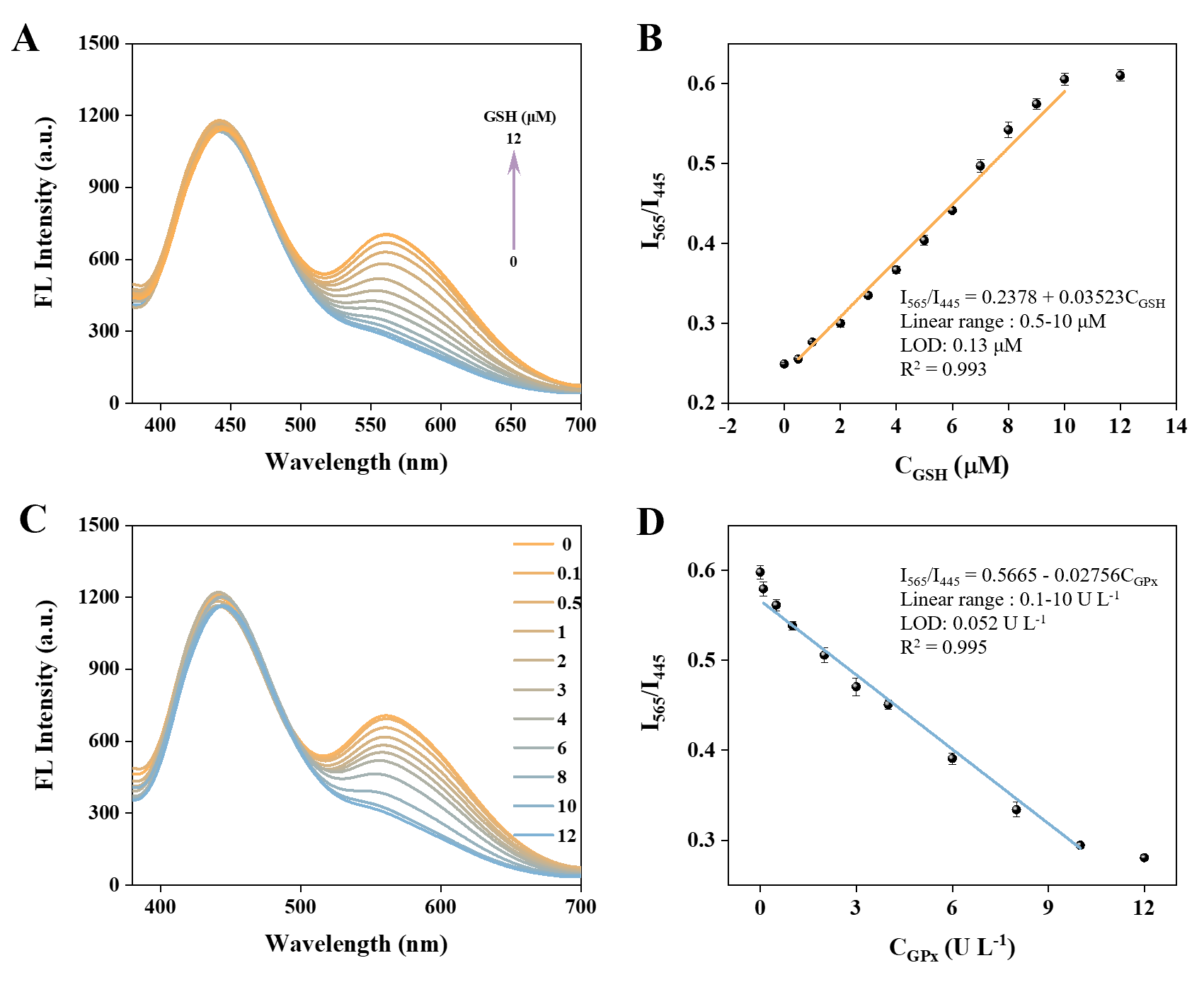


**Figure S21.** (A) Fluorescence spectra of Au-ZIF-8@CDs/Cu^2+^ in the presence of various concentrations of GSH. (B) Plot of I_565_/I_445_ versus GSH concentration. (C) Fluorescence spectra of Au-ZIF-8@CDs/Cu^2+^/GSH/H_2_O_2_ with various concentration of GPx. (D) The lineal plot between I_565_/I_445_ and GPx activities.


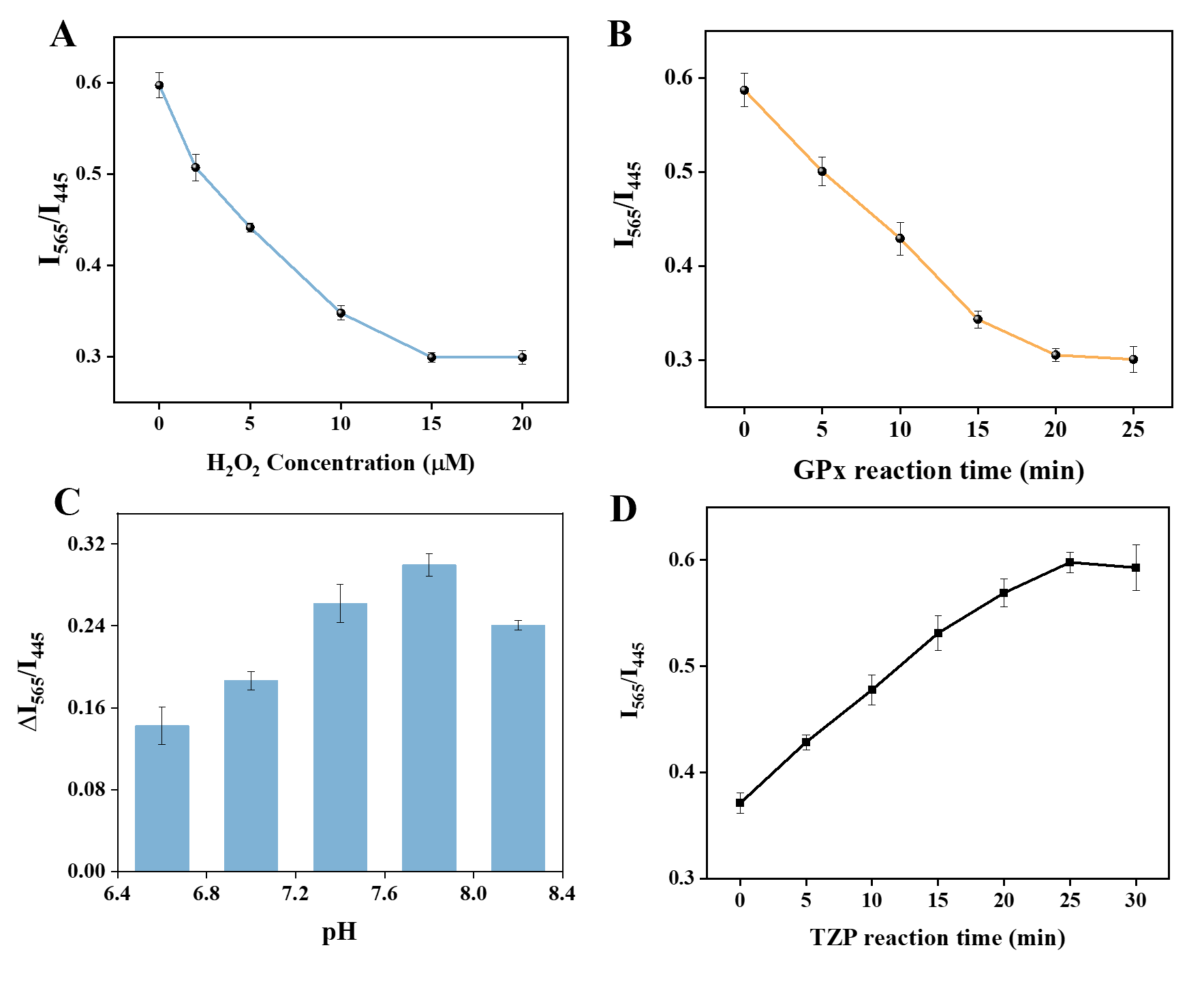


**Figure S22.** The optimization of (A) H_2_O_2_ concentration, (B) GPx incubation time, (C) pH, and (D) TZP incubation time for TZP detection.

**Figure S23.** The reproducibility of Au-ZIF@CDs-based platform toward TZP (100 ng mL^−1^).


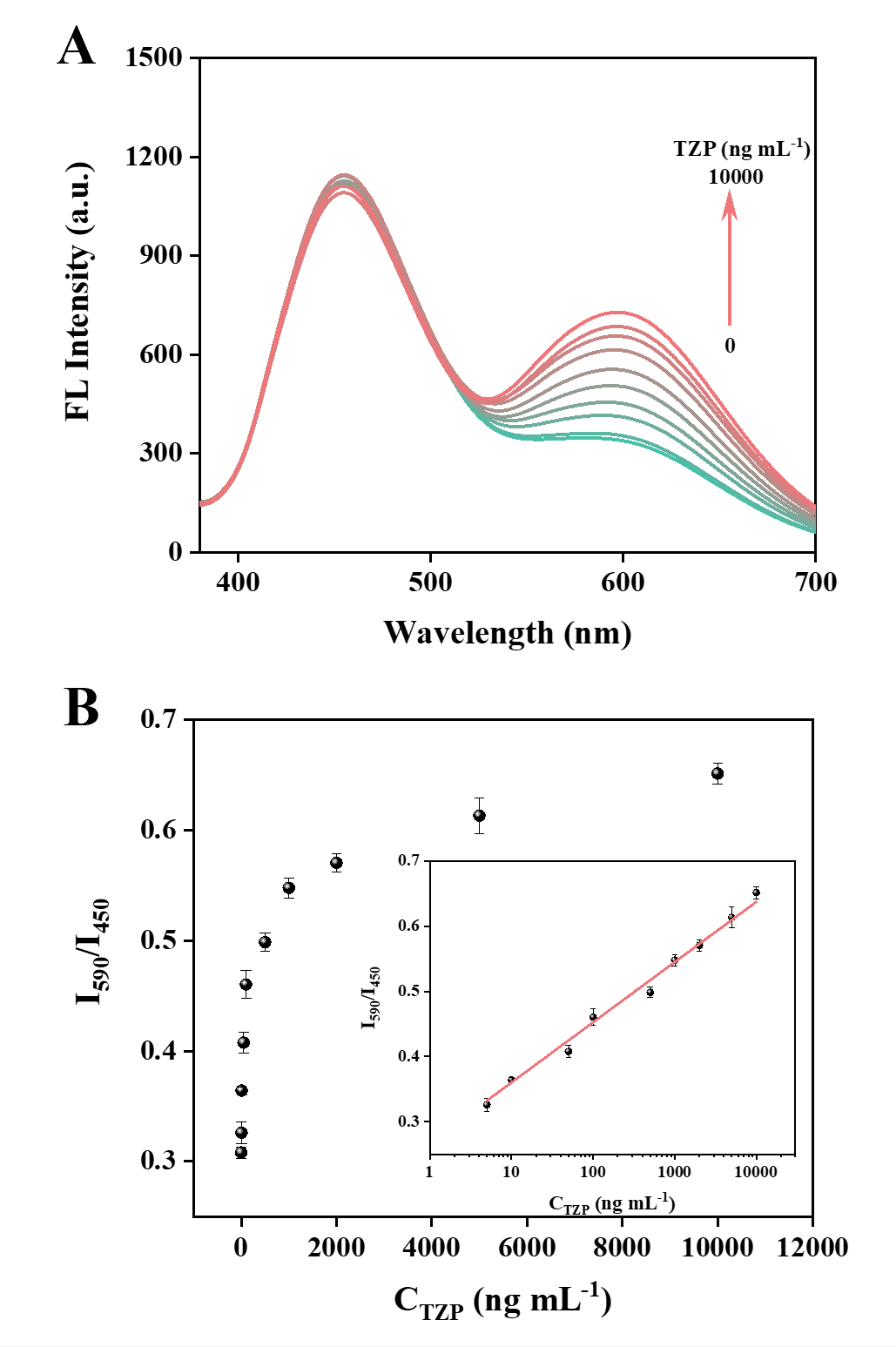


**Figure S24.** (A) Fluorescence emission spectra of Au-ZIF@CDs/Cu^2+^/GSH/H_2_O_2_/GPx system with varied TZP concentrations. (B) Fluorescence emission intensity ratio I_590_/I_450_ of Au-ZIF@CDs/Cu^2+^/GSH/H_2_O_2_/GPx system with varied TZP concentrations, with the linear equation: I_590_/I_450_ = 0.2678 + 0.09244LogC_TZP_ (R^2^ = 0.991) and the LOD of 4.2 ng mL^-1^.


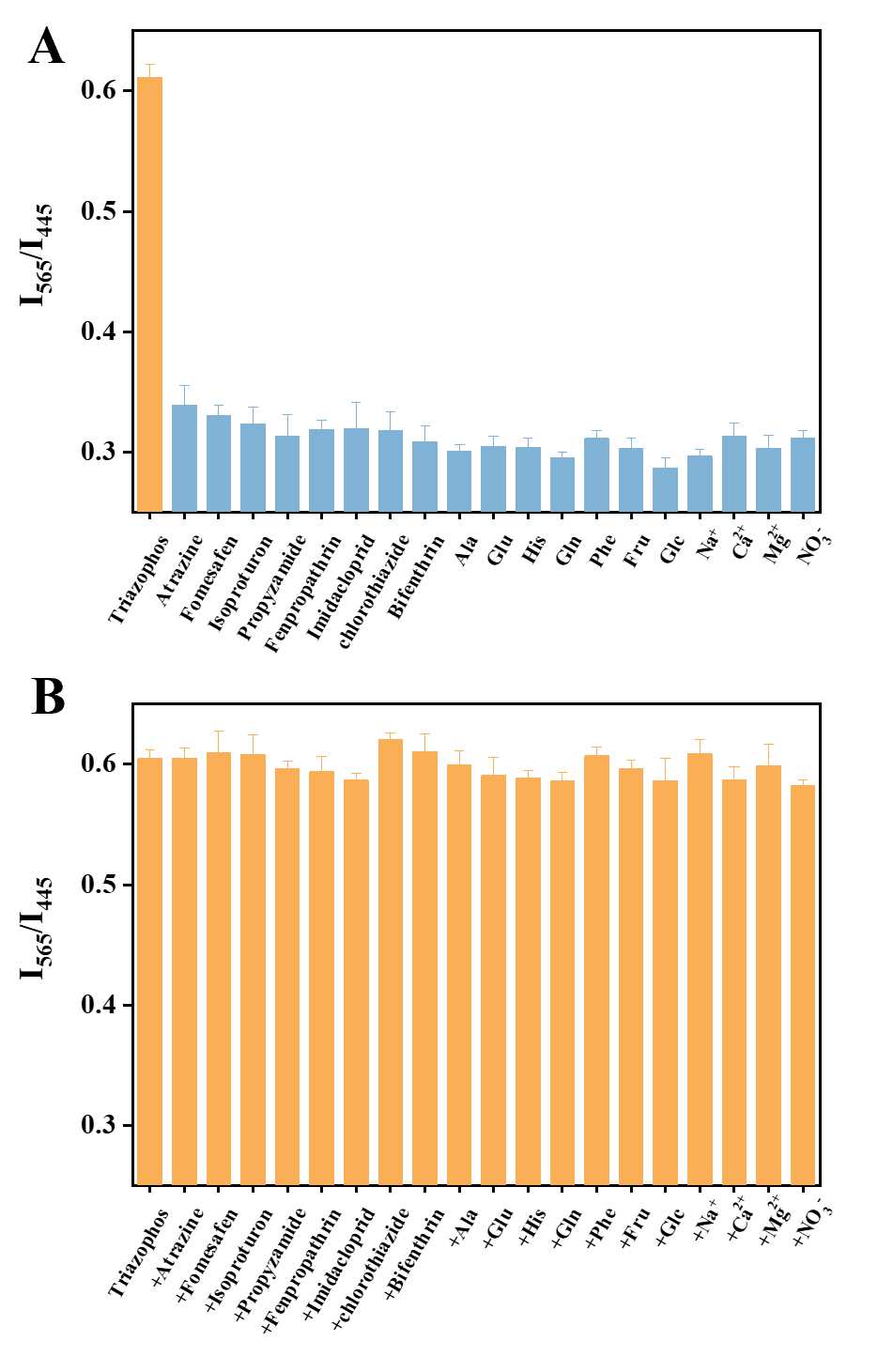


**Figure S25. (A)** Selectivity (B) Anti-interference ability of Au-ZIF@CDs-based method for TZP.


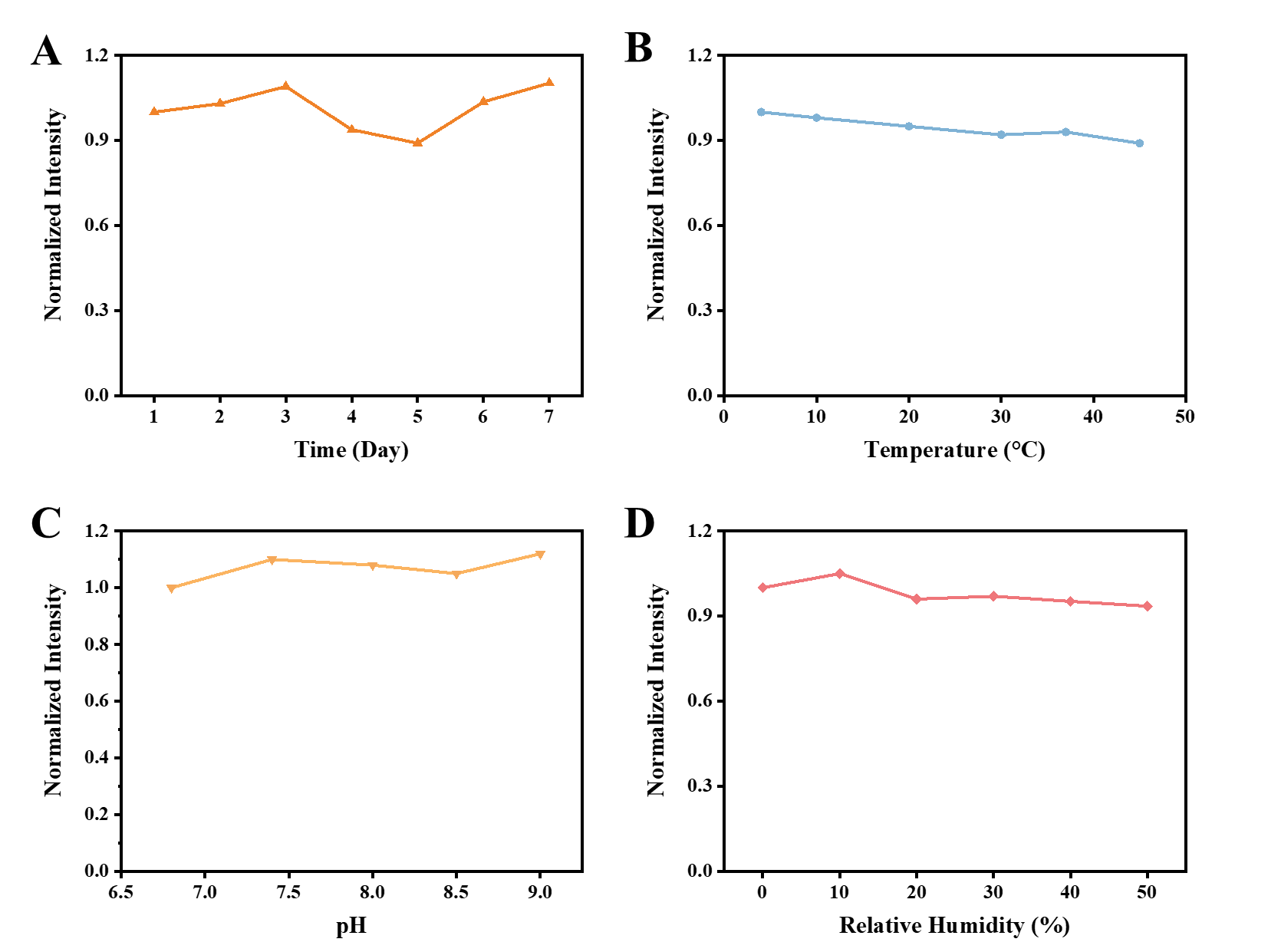


**Figure S26.** The stability of the hydrogel-based sensors under different conditions, including (A) time, (B) temperature, (C) pH, and (D) humidity.


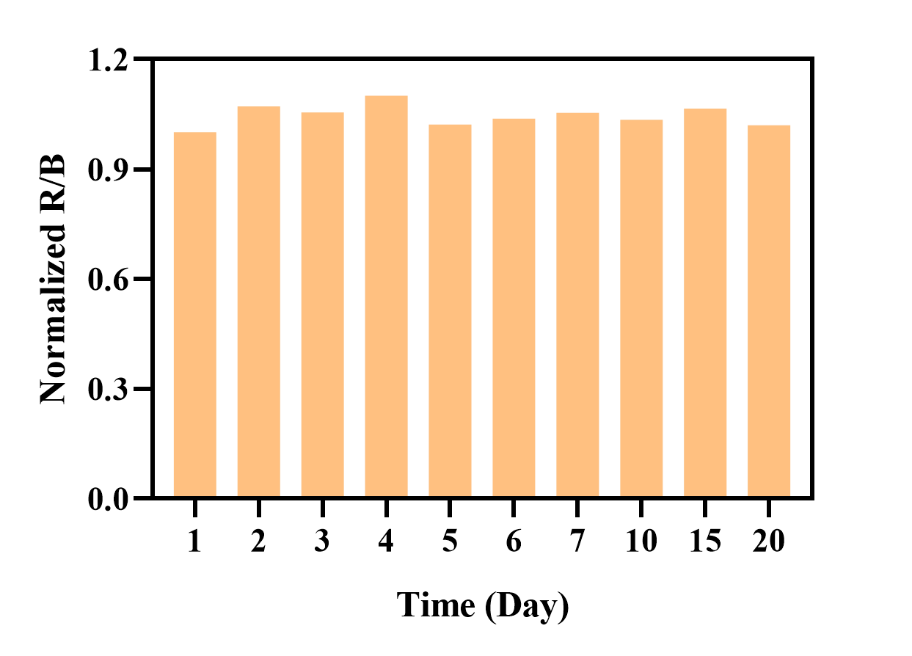


**Figure S27.** The hydrogel-based sensors stability while sensing triazophos (100 ng mL^−1^).


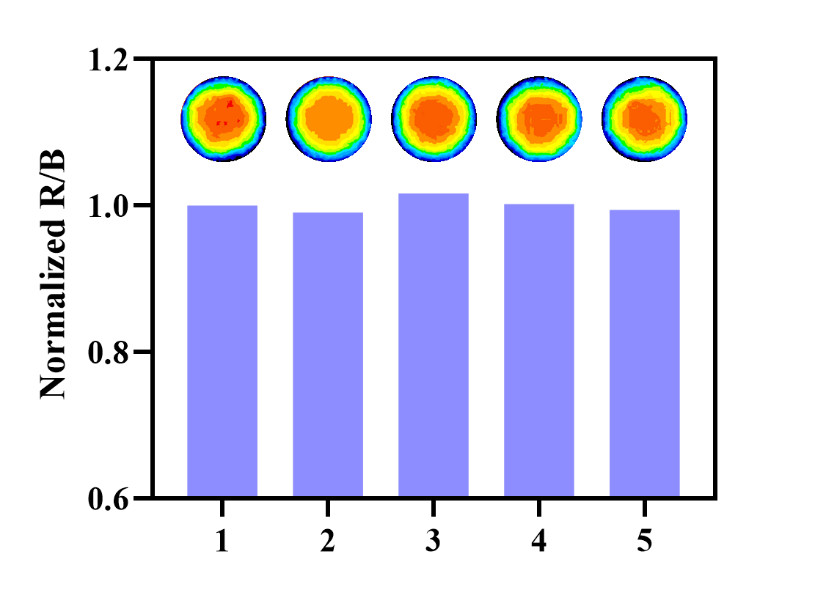


**Figure S28.** The reproducibility of hydrogel-based sensors toward triazophos (100 ng/mL).


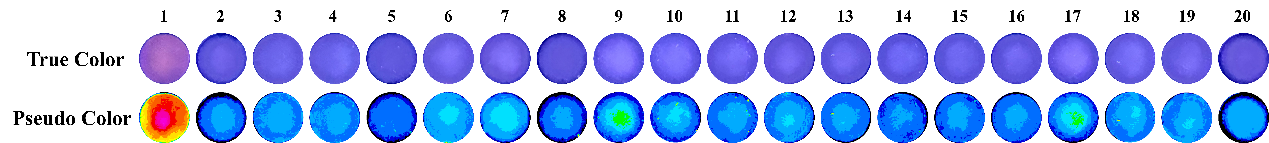


**Figure S29.** Selectivity performance of hydrogel discs. (1) Triazophos, (2) Atrazine, (3) Fomesafen, (4) Isoproturon, (5)Propyzamide, (6) Fenpropathrin, (7) Imidacloprid, (8) Chlorothiazide, (9) Bifenthrin, (10) Alanine, (11) Glutamate, (12) Histidine, (13) Glutamine, (14) Phenylalanine, (15) Fructose, (16) Glucose, (17) Na^+^, (18) Ca^2+^, (19) Mg^2+^, (20) NO_3_^-^.


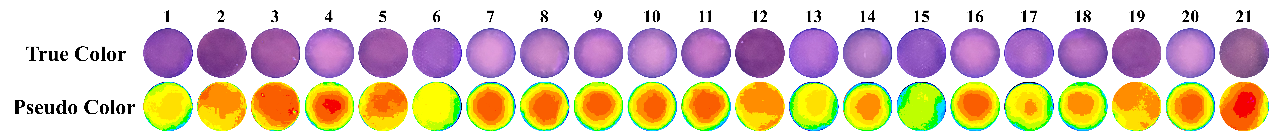


**Figure S30.** Anti-interference performance of hydrogel discs. (1) Blank, (2) Triazophos, (3) Atrazine, (4) Fomesafen, (5) Isoproturon, (6) Propyzamide, (7) Fenpropathrin, (8) Imidacloprid, (9) Chlorothiazide, (10) Bifenthrin, (11) Alanine, (12) Glutamate, (13) Histidine, (14) Glutamine, (15) Phenylalanine, (16) Fructose, (17) Glucose, (18) Na^+^, (19) Ca^2+^, (20) Mg^2+^, (21) NO_3_^-^.


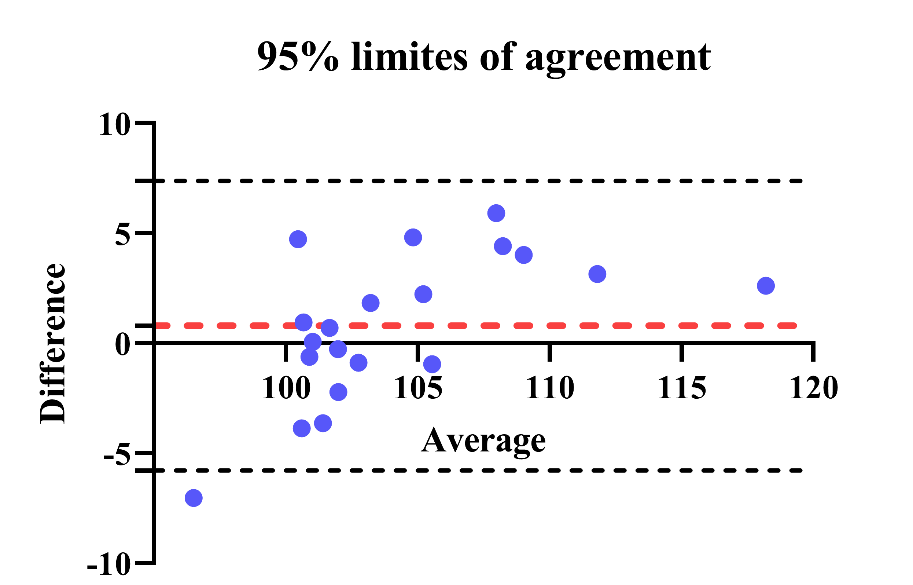


**Figure S31.** Bland-Altman analysis was used to evaluate the recovery agreement between HPLC and the established biosensor. Bland-Altman analysis of HPLC and established biosensor agreement demonstrated that the 95% limits of agreement ranged from -5.792 to 7.373. The biosensor results for analyzing pesticides showed acceptable agreement with HPLC installed in the standard laboratory of the testing agency, demonstrating the good reliability and feasibility of biosensors for monitoring pesticide residues.


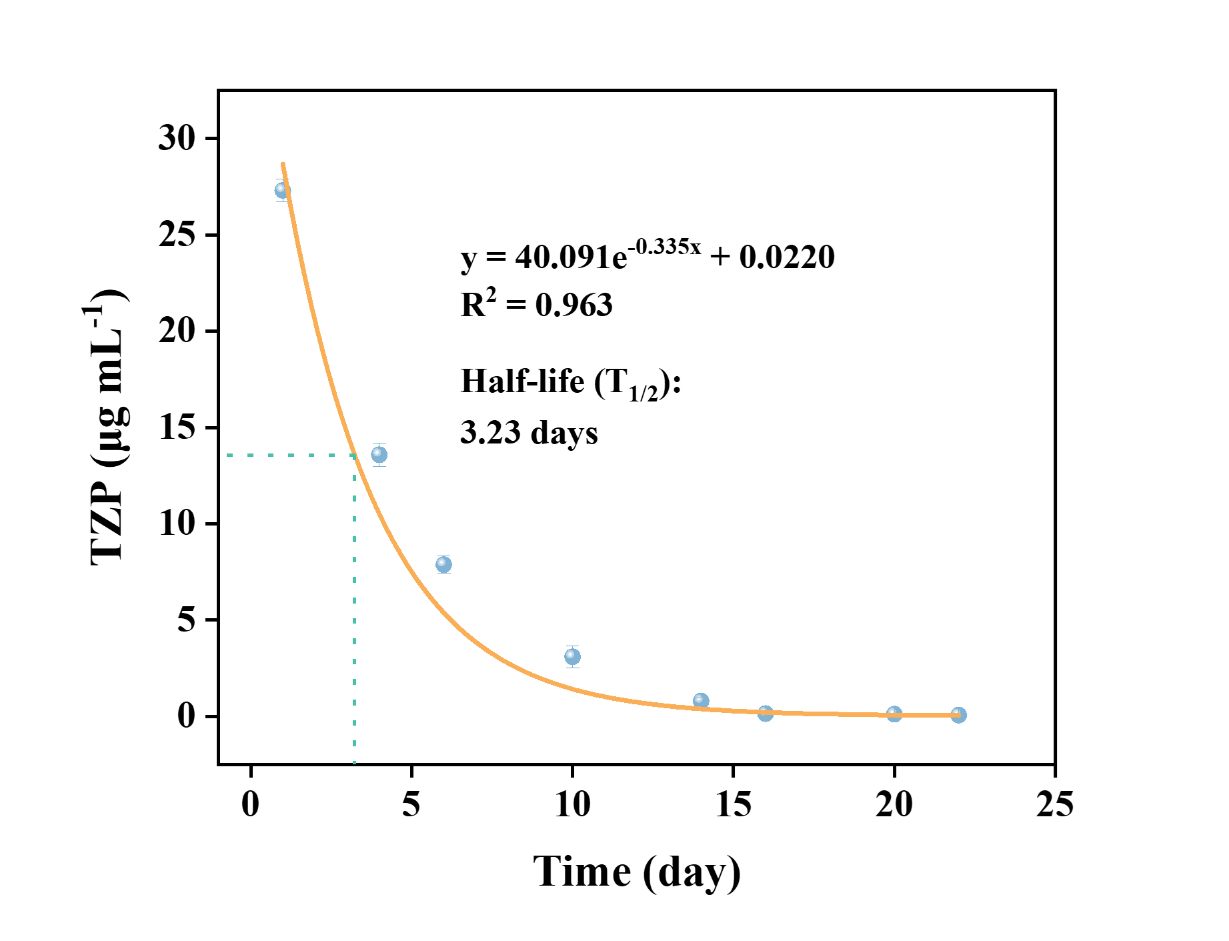


**Figure S32.** Degradation monitoring of TZP in the leaves within 22 days using HPLC. The half-life (3.23 days) was similar to that of Au-ZIF@CDs-based method.

**Table S1.** Fitting parameters for the AuNCs, Au-ZIF, Au-ZIF@CDs, Au-ZIF@CDs+ Cu^2+^, and Au-ZIF@CDs+Cu^2+^+GSH.

| Sample | *τ*_1_ (μs) | B_1_ | *τ*_2_ (μs) | B_2_ | *τ_A_* (μs) |
| --- | --- | --- | --- | --- | --- |
| AuNCs  Au-ZIF  Au-ZIF@CDs  Au-ZIF@CDs/Cu^2+^  Au-ZIF@CDs/Cu^2+^/GSH | 0.7839  2.3519  1.7828  0.4699  2.0065 | 3155.5  1822.0  2003.5  485.2  1405.3 | 4.3987  11.6451  10.5678  6.3914  10.433 | 824.3  1110.1  1389.4  114.515  655.3 | 2.93  9.33  8.85  4.98  7.97 |

**Table S2.** Comparison of performance of different OPs probing strategy.

| Mehod | Linear range (ng·mL^-1^) | LOD (ng·mL^-1^) | Time (min) | Reference |
| --- | --- | --- | --- | --- |
| Paper-based Electrochemistry | 100-1000 | 50 | / | ^9^ |
| Electrochemical sensor | 124-1000 | 125 | 20 | ^10^ |
| TiO_2_-based photoelectrochemistry | 110-2763 | 2.2 | / | ^11^ |
| Chip-based colorimetric sensor | 0-8000 | 100 | 140 | ^12^ |
| AuNPs-based colorimetric sensor | 11.3-123.75 | 11.3 | 185 | ^13^ |
| MnO_2_-based fluorescence sensor | 2.5-300 | 0.73 | 35 | ^14^ |
| QD@SiO2@NBD@MIPs-based fluorescence sensor | 100-1500 | 100 | / | ^15^ |
| CDs-based fluorescence sensor | 100-4500 | 23 | 30 | ^16^ |
| MnMOF-based fluorescence sensor | 1-10000 | 0.6 | 60 | ^17^ |
| Fe_3_O_4_@COF-Au-based Chemiluminescence sensor | 5-300 | 1 | 60 | ^18^ |
| rQDs@SiO2@gQDs-based fluorescence sensor | 0-1690 | 0.475 | 75 | ^19^ |
| UCNPs@ZIF@PDA hydrogel disc-based fluorescence sensor | 20-100000 | 20 | 85 | ^20^ |
| Au-ZIF@CDs hydrogel-based fluorescence sensor | 0.2-3000 | 0.09 | 45 | This work |

**Table S3.** Determination of TZP in spiked samples using hydrogel-based sensors.

| Sample | Detected (μg L^-1^, μg kg^-1^) | Added (μg L^-1^, μg kg^-1^) | Founded (μg L^-1^, μg kg^-1^) | | | Average recovery (%) | RSD (%) |
| --- | --- | --- | --- | --- | --- | --- | --- |
|  |  |  | 1 | 2 | 3 |  |  |
| Apple juice | Not detected | 1 | 1.03 | 1.05 | 1.09 | 105.67 | 2.89 |
|  |  | 10 | 9.12 | 10.07 | 9.54 | 95.77 | 4.97 |
|  |  | 100 | 109.62 | 110.43 | 114.81 | 111.62 | 2.50 |
| Cabbage | Not detected | 1 | 1.00 | 1.03 | 1.01 | 101.33 | 1.51 |
|  |  | 10 | 10.30 | 9.38 | 9.34 | 96.73 | 5.61 |
|  |  | 100 | 95.75 | 96.37 | 106.41 | 99.51 | 6.00 |
| Cucumber | Not detected | 1 | 0.96 | 0.93 | 0.94 | 94.33 | 1.62 |
|  |  | 10 | 9.05 | 9.59 | 8.81 | 91.50 | 4.37 |
|  |  | 100 | 89.44 | 101.84 | 93.90 | 95.06 | 6.61 |
| Soil | Not detected | 1 | 0.93 | 0.88 | 0.96 | 92.33 | 4.38 |
|  |  | 10 | 9.91 | 9.50 | 10.30 | 99.03 | 4.04 |
|  |  | 100 | 102.33 | 92.99 | 96.68 | 97.33 | 4.83 |

**References**

(1) Luo, Z.; Yuan, X.; Yu, Y.; Zhang, Q.; Leong, D. T.; Lee, J. Y.; Xie, J. From Aggregation-Induced Emission of Au(I)–Thiolate Complexes to Ultrabright Au(0)@Au(I)–Thiolate Core–Shell Nanoclusters. *Journal of the American Chemical Society* **2012**, *134* (40), 16662-16670. DOI: 10.1021/ja306199p.

(2) Zhang, J.; Lu, X.; Tang, D.; Wu, S.; Hou, X.; Liu, J.; Wu, P. Phosphorescent Carbon Dots for Highly Efficient Oxygen Photosensitization and as Photo-oxidative Nanozymes. *ACS Applied Materials & Interfaces* **2018**, *10* (47), 40808-40814. DOI: 10.1021/acsami.8b15318.

(3) Zhao, D.; Dong, C.-L.; Wang, B.; Chen, C.; Huang, Y.-C.; Diao, Z.; Li, S.; Guo, L.; Shen, S. Synergy of Dopants and Defects in Graphitic Carbon Nitride with Exceptionally Modulated Band Structures for Efficient Photocatalytic Oxygen Evolution. *Advanced Materials* **2019**, *31* (43), 1903545. DOI: https://doi.org/10.1002/adma.201903545 (acccessed 2024/06/25).

(4) Delley, B. An all‐electron numerical method for solving the local density functional for polyatomic molecules. *The Journal of Chemical Physics* **1990**, *92* (1), 508-517. DOI: 10.1063/1.458452 (acccessed 7/8/2024).

(5) Delley, B. From molecules to solids with the DMol3 approach. *The Journal of Chemical Physics* **2000**, *113* (18), 7756-7764. DOI: 10.1063/1.1316015 (acccessed 7/8/2024).

(6) Tian, D.; Zhang, H.; Zhao, J. Structure and structural evolution of Agn (n=3–22) clusters using a genetic algorithm and density functional theory method. *Solid State Communications* **2007**, *144* (3), 174-179. DOI: https://doi.org/10.1016/j.ssc.2007.05.020.

(7) Hammer, B.; Hansen, L. B.; Nørskov, J. K. Improved adsorption energetics within density-functional theory using revised Perdew-Burke-Ernzerhof functionals. *Physical Review B* **1999**, *59* (11), 7413-7421. DOI: 10.1103/PhysRevB.59.7413.

(8) Björkman, T.; Gulans, A.; Krasheninnikov, A. V.; Nieminen, R. M. van der Waals Bonding in Layered Compounds from Advanced Density-Functional First-Principles Calculations. *Physical Review Letters* **2012**, *108* (23), 235502. DOI: 10.1103/PhysRevLett.108.235502.

(9) Arduini, F.; Cinti, S.; Caratelli, V.; Amendola, L.; Palleschi, G.; Moscone, D. Origami multiple paper-based electrochemical biosensors for pesticide detection. *Biosensors and Bioelectronics* **2019**, *126*, 346-354. DOI: https://doi.org/10.1016/j.bios.2018.10.014.

(10) Zhang, Y.; Arugula, M. A.; Wales, M.; Wild, J.; Simonian, A. L. A novel layer-by-layer assembled multi-enzyme/CNT biosensor for discriminative detection between organophosphorus and non-organophosphrus pesticides. *Biosensors and Bioelectronics* **2015**, *67*, 287-295. DOI: https://doi.org/10.1016/j.bios.2014.08.036.

(11) Shi, H.; Zhao, G.; Liu, M.; Zhu, Z. A novel photoelectrochemical sensor based on molecularly imprinted polymer modified TiO2 nanotubes and its highly selective detection of 2,4-dichlorophenoxyacetic acid. *Electrochemistry Communications* **2011**, *13* (12), 1404-1407. DOI: https://doi.org/10.1016/j.elecom.2011.08.022.

(12) Meng, X.; Schultz, C. W.; Cui, C.; Li, X.; Yu, H.-Z. On-site chip-based colorimetric quantitation of organophosphorus pesticides using an office scanner. *Sensors and Actuators B: Chemical* **2015**, *215*, 577-583. DOI: https://doi.org/10.1016/j.snb.2015.04.011.

(13) Sun, J.; Guo, L.; Bao, Y.; Xie, J. A simple, label-free AuNPs-based colorimetric ultrasensitive detection of nerve agents and highly toxic organophosphate pesticide. *Biosensors and Bioelectronics* **2011**, *28* (1), 152-157. DOI: https://doi.org/10.1016/j.bios.2011.07.012.

(14) Chen, J.; Chen, X.; Zhao, J.; Liu, S.; Chi, Z. Instrument-free and visual detection of organophosphorus pesticide using a smartphone by coupling aggregation-induced emission nanoparticle and two-dimension MnO2 nanoflake. *Biosensors and Bioelectronics* **2020**, *170*, 112668. DOI: https://doi.org/10.1016/j.bios.2020.112668.

(15) Wang, X.; Yu, J.; Wu, X.; Fu, J.; Kang, Q.; Shen, D.; Li, J.; Chen, L. A molecular imprinting-based turn-on Ratiometric fluorescence sensor for highly selective and sensitive detection of 2,4-dichlorophenoxyacetic acid (2,4-D). *Biosensors and Bioelectronics* **2016**, *81*, 438-444. DOI: https://doi.org/10.1016/j.bios.2016.03.031.

(16) Su, D.; Han, X.; Yan, X.; Jin, R.; Li, H.; Kong, D.; Gao, H.; Liu, F.; Sun, P.; Lu, G. Smartphone-Assisted Robust Sensing Platform for On-Site Quantitation of 2,4-Dichlorophenoxyacetic Acid Using Red Emissive Carbon Dots. *Analytical Chemistry* **2020**, *92* (18), 12716-12724. DOI: 10.1021/acs.analchem.0c03275.

(17) Liu, J.; Mo, Y. Y.; Zhang, H.; Tang, J.; Bao, H.; Wei, L.; Yang, H. Target-Responsive Metal–Organic Framework Nanosystem with Synergetic Sensitive Detection and Controllable Degradation against the Pesticide Triazophos in Contaminated Samples for Environment Assessment and Food Safety. *ACS Applied Materials & Interfaces* **2023**, *15* (19), 23783-23791. DOI: 10.1021/acsami.3c03248.

(18) Ma, Y.; Zhao, Y.; Xu, X.; Ding, S.; Li, Y. Magnetic covalent organic framework immobilized gold nanoparticles with high-efficiency catalytic performance for chemiluminescent detection of pesticide triazophos. *Talanta* **2021**, *235*, 122798. DOI: https://doi.org/10.1016/j.talanta.2021.122798.

(19) Wei, D.; Wang, Y.; Zhu, N.; Xiao, J.; Li, X.; Xu, T.; Hu, X.; Zhang, Z.; Yin, D. A Lab-in-a-Syringe Device Integrated with a Smartphone Platform: Colorimetric and Fluorescent Dual-Mode Signals for On-Site Detection of Organophosphorus Pesticides. *ACS Applied Materials & Interfaces* **2021**, *13* (41), 48643-48652. DOI: 10.1021/acsami.1c13273.

(20) Yan, X.; Ma, Y.; Lu, Y.; Su, C.; Liu, X.; Li, H.; Lu, G.; Sun, P. Zeolitic Imidazolate-Framework-Engineered Heterointerface Catalysis for the Construction of Plant-Wearable Sensors. *Advanced Materials* **2024**, *36* (16), 2311144. DOI: https://doi.org/10.1002/adma.202311144 (acccessed 2024/06/25).
